# From proteome-wide Mendelian randomization and multi-omics integration to functional validation: TGFB3 as a prioritized candidate in gastric adenocarcinoma

**DOI:** 10.3389/fonc.2026.1883227

**Published:** 2026-07-06

**Authors:** Luming Zhao, Chenxi Mao, Yimeng Xu, Kangjie Zhou, Mingtong Liang, Yiqian Han, Jingzhou Zhang, Yidong Hong, Nan Hu, Fenglei Wu

**Affiliations:** 1 Department of Oncology, The Affiliated Lianyungang Hospital of Xuzhou Medical University/The First People’s Hospital of Lianyungang, Lianyungang, China; 2 Lianyungang Clinical College of Nanjing Medical University/The First People’s Hospital of Lianyungang, Lianyungang, China

**Keywords:** drug screening, gastric cancer, Mendelian randomization, plasma proteomics, transcriptome analysis

## Abstract

**Introduction:**

Gastric adenocarcinoma lacks robust circulating biomarkers and tractable targets. We assessed whether genetically predicted plasma protein levels influence disease risk and sought druggable candidates using a proteome-wide Mendelian randomization (MR) framework.

**Methods:**

We integrated deCODE plasma protein quantitative trait loci (pQTLs) with gastric cancer GWAS in a proteome-wide two-sample MR framework to identify circulating proteins causally associated with gastric cancer. Protein–protein interaction topology was used to prioritize eight hub proteins, which were further evaluated in an eight-gene artificial neural network (ANN) classifier. Single-cell and spatial transcriptomics, together with multiplex immunofluorescence, mapped hub-gene expression across tumor, stromal and immune compartments. Finally, we focused on TGFB3 as a genetically and spatially prioritized target, combining in silico ligand screening with *in vitro* perturbation of the TGFB3–PI3K survival axis using proflavine hemisulfate as a chemical probe.

**Results:**

Proteome-wide MR highlighted 29 circulating proteins with putative causal effects on gastric cancer, of which eight (ERBB3, LGR4, BMP4, CD248, MGP, TGFB3, GRP and ETS2) occupied central positions in the interaction network. The corresponding eight-gene ANN showed robust discrimination between gastric cancer and non-tumor tissue across multiple cohorts and improved diagnostic performance beyond clinical variables. Single-cell, spatial and multiplex immunofluorescence analyses localized these hubs to epithelial, fibroblast and endothelial compartments, with TGFB3 enriched at tumor–stroma interfaces and associated with poor survival. Virtual screening nominated TGFB3-binding ligands and proflavine hemisulfate formed a stable complex with TGFB3 in silico while attenuating TGFB3-driven proliferation, migration and PI3K-dependent survival signaling in AGS cells.

**Conclusion:**

Our proteome-wide MR framework identifies genetically supported circulating proteins that contribute to gastric carcinogenesis and converges on TGFB3 as a tractable stromal signaling axis. Proflavine hemisulfate functions as a mechanistically informative chemical probe of TGFB3–PI3K survival signaling in gastric cancer cells, providing a starting scaffold for future TGFB3-targeted therapeutic strategies.

## 1 Introduction

Gastric cancer remains one of the most lethal malignancies worldwide, ranking among the five most commonly diagnosed cancers and the five leading causes of cancer-related mortality, with nearly one million new cases and more than 660,000 deaths estimated globally in 2022 (1, 2). This high mortality burden is driven in part by delayed diagnosis, marked biological heterogeneity, and limited durability of treatment response in advanced disease. Current management is strongly stage- and biomarker-dependent. For localized or resectable gastric adenocarcinoma, curative-intent treatment generally relies on gastrectomy with adequate lymphadenectomy, often integrated with perioperative chemotherapy, adjuvant chemotherapy, or chemoradiotherapy according to disease stage, surgical context, and regional practice (3). For unresectable, recurrent, or metastatic disease, systemic therapy has progressively shifted toward molecularly stratified treatment, including HER2-directed therapy, antiangiogenic agents, immune checkpoint blockade, and biomarker-guided strategies based on HER2, PD-L1, MSI/MMR, and CLDN18.2 status (3, 4). Nevertheless, only a subset of patients derives sustained benefit from these approaches. Conventional serum biomarkers have limited sensitivity and specificity, and established molecular markers do not fully capture the biological heterogeneity of gastric cancer. These limitations underscore the need to identify additional circulating biomarkers and biologically tractable protein programs linked to gastric cancer risk and tumor behavior.

At the molecular level, gastric tumorigenesis is shaped by coordinated alterations in epithelial growth, stromal remodeling, angiogenesis, immune regulation, and extracellular matrix organization. Comprehensive molecular profiling has classified gastric adenocarcinoma into biologically distinct subtypes, including Epstein–Barr virus-positive tumors, microsatellite instability tumors, genomically stable tumors, and chromosomal instability tumors (5, 6). Across these molecular contexts, dysregulated signaling through ERBB/MAPK, PI3K–AKT, TGF-β, WNT–β-catenin, VEGF-related angiogenic pathways, and immune checkpoint programs contributes to tumor initiation, progression, and therapeutic adaptation (1, 5, 6). However, many pathway-level observations are derived from tumor tissue or cross-sectional expression studies and are therefore susceptible to confounding by tumor burden, treatment exposure, cellular composition, and reverse causation. A major unresolved challenge is to connect systemic biomarker signals with genetically anchored disease susceptibility and tumor-resolved biological context.

Plasma proteins provide a particularly informative interface between systemic physiology and local tumor biology. They may originate from active secretion, extracellular matrix remodeling, immune and stromal signaling, or tumor-cell shedding, and therefore have potential value as biomarkers, mediators, or therapeutic hypotheses (7, 8). Observational proteomic studies have identified numerous disease-associated circulating proteins, but these associations do not readily distinguish disease causes from consequences and may be influenced by residual or unmeasured confounding (9). Protein quantitative trait loci (pQTLs) now enable Mendelian randomization (MR) analyses that use germline genetic variation as instrumental variables to estimate associations between genetically predicted protein abundance and disease risk (10–12). Because germline variants are fixed before disease onset, MR can reduce confounding and reverse causation relative to conventional observational designs. Importantly, however, MR should be interpreted as evidence of genetically supported protein–disease association rather than direct proof of protein-level functional causality within tumor tissue. Proteome-wide MR has been increasingly applied to prioritize circulating protein candidates for biomarker discovery and target nomination across multiple malignancies, including colorectal, breast, pancreatic, and lung cancers (13–16).

In this study, we integrated cis- and trans-pQTL data with gastric cancer GWAS summary statistics to perform a proteome-wide two-sample MR analysis of circulating plasma proteins. Proteins associated with gastric cancer risk were then contextualized through sensitivity analyses, independent GWAS replication, exploratory functional annotation, and protein–protein interaction network prioritization. A compact set of network-prioritized candidates was further evaluated using an artificial neural network classifier with SHAP-based interpretability, allowing assessment of their nonlinear predictive contributions while avoiding mechanistic overinterpretation of model features. To connect genetically supported circulating protein signals with local tumor biology, we mapped the corresponding genes across single-cell RNA sequencing and spatial transcriptomic datasets, assessed their prognostic associations, and performed multiplex immunofluorescence staining in an independent gastric cancer tissue cohort. Finally, TGFB3 was selected as a convergent candidate for focused chemical-probe interrogation using virtual screening, molecular docking, molecular dynamics simulation, and *in vitro* perturbation assays in gastric cancer cells. Together, this multi-layered framework links germline genetics, circulating protein biology, tumor-resolved transcriptomics, spatial protein localization, and cellular perturbation experiments to nominate candidate plasma-protein programs for further mechanistic validation in gastric adenocarcinoma.

## 2 Methods and materials

### 2.1 Study datasets and selection of genetic instrumental variables

Genome-wide association study (GWAS) summary statistics for gastric cancer were obtained from the IEU GWAS database (https://gwas.mrcieu.ac.uk; dataset code ebi-a-GCST90435577). This dataset comprised 393,926 individuals of European ancestry (393,372 controls and 554 cases) and included 35,642,134 single-nucleotide polymorphisms (SNPs). To assess the robustness and generalizability of the findings, we additionally used two independent gastric cancer GWAS datasets in downstream replication analyses: a larger European GWAS from the GWAS Catalog (ebi-a-GCST90018849; 1,029 cases and 290,685 controls) and an East-Asian GWAS from BioBank Japan (bbj-a-119; 6,563 cases and 195,745 controls). Plasma proteome GWAS data were sourced from the deCODE database (https://www.decode.com), based on Ferkingstad et al. (12), who analyzed plasma protein levels—measured via 4,907 aptamers—in 35,559 Icelandic participants. As the exposure and outcome GWAS originated from distinct populations, sample overlap is unlikely. All procedures conformed to the Declaration of Helsinki (2013 revision).Instrumental variables (IVs) were selected according to three criteria (17): (1) robust association with the exposure (plasma protein expression); (2) no association with potential confounders; and (3) effect on the outcome solely via the exposure. SNPs were chosen as IVs due to their lifetime stability, random segregation at gametogenesis, and ease of genotyping (18). Both cis- and trans-pQTLs were considered. SNPs meeting P < 5 × 10^-8^ were subjected to linkage disequilibrium (LD) clumping (r² < 0.001 within 10 Mb) using the TwoSampleMR R package, and only those with F-statistics > 10 were retained to mitigate weak-instrument bias; detailed F values for each protein are provided in **Supplementary Table 1**. Where exposure SNPs were absent in the outcome dataset, proxy variants in strong LD (r² > 0.8) were used. To ensure the correct causal direction, we further applied Steiger filtering by comparing the variance in the exposure and outcome explained by each SNP and retaining only variants with stronger associations with protein levels than with gastric cancer; SNPs failing this criterion were excluded (**Supplementary Table 2**).

### 2.2 Proteome-wide MR, power evaluation and sensitivity analyses

We conducted two-sample MR analyses for 4,907 plasma proteins to assess their causal associations with gastric cancer. All analyses were performed in R (v4.3.2) using the TwoSampleMR package (19). The inverse-variance weighted (IVW) method served as the primary estimator, regressing SNP–outcome on SNP–exposure effects with the intercept fixed at zero (20). Proteins instrumented by a single SNP were analyzed via the Wald ratio. To control false positives, P values were adjusted by the Benjamini–Hochberg false discovery rate (FDR) procedure, and proteins with FDR-adjusted P < 0.05 were considered significant.

For plasma proteins included in MR analysis, we formally assessed instrument strength and statistical power. Protein-specific R² values were derived from the SNP–exposure summary statistics, and F-statistics were computed for each instrument. These quantities, together with the effective sample size of the corresponding gastric cancer GWAS and anticipated effect sizes in the range of odds ratio (OR) = 1.3–1.5 at α = 0.05, were used to estimate MR power using standard analytical formulae (**Supplementary Table 3**).

Robustness was evaluated through sensitivity analyses (21): horizontal pleiotropy was tested by the MR–Egger intercept (a nonzero intercept indicates directional pleiotropy) (22), and heterogeneity across instruments was quantified by Cochran’s Q statistic (higher Q denotes greater heterogeneity) (23). MR-PRESSO was applied to identify and, where appropriate, correct for outlier variants with disproportionate influence on the MR estimates (**Supplementary Table 4**). Leave-one-out analyses were performed by iteratively excluding each SNP in turn to assess whether the causal estimates were driven by any single instrument.

### 2.3 Functional and pathway enrichment analysis

Gene Ontology (GO) annotation and Kyoto Encyclopedia of Genes and Genomes (KEGG) pathway enrichment analyses of gastric cancer–causally associated plasma proteins were performed using the DAVID online resource (https://davidbioinformatics.nih.gov/). These analyses elucidated the potential involvement of key proteins in biological processes, molecular functions and cellular components, as well as the signaling pathways in which they participate. To present the results more intuitively, we utilized the online data analysis and visualization platform at https://www.bioinformatics.com.cn, which provides streamlined tools for data display and enhances both interpretability and graphical presentation.

### 2.4 Construction of the PPI network and network-prioritized genes in gastric cancer

Protein–protein interaction (PPI) analysis of gastric cancer–causally associated plasma proteins was conducted using the STRING database (https://cn.string-db.org/) with a minimum interaction confidence score of 0.4. The resultant PPI network was imported into Cytoscape v3.10.3 (https://cytoscape.org/) for visualization and calculation of node metrics. Node centrality was quantified using the cytoHubba plugin (v0.1). We *a priori* focused on two complementary measures: degree centrality and Edge Percolated Component (EPC). Degree centrality reflects the number of direct interaction partners of a protein and therefore identifies highly connected nodes that may serve as information hubs within the network. EPC, in contrast, evaluates the stability of a node’s connectivity under iterative edge percolation and highlights proteins that reside in densely interconnected and topologically robust sub-networks. For each metric, all nodes were ranked in descending order, and the top ten proteins were extracted according to degree and EPC, respectively. To obtain a conservative set of network-prioritized genes that are both highly connected and embedded in resilient interaction modules, we defined network-prioritized genes as the intersection of these two top-ten lists.

### 2.5 Artificial neural network modeling

To integrate the expression patterns of eight MR-prioritized hub genes (ERBB3, LGR4, BMP4, CD248, MGP, TGFB3, GRP, and ETS2) for tumor discrimination, we implemented a feed-forward multilayer perceptron (MLP) in R using the neuralnet and NeuralNetTools packages (24). The ANN was selected because nonlinear relationships among the eight genes may not be fully captured by linear classifiers; however, we did not assume *a priori* that ANN would necessarily outperform simpler or more interpretable models. Therefore, the ANN was benchmarked against logistic regression, ridge logistic regression, LASSO logistic regression, and random forest using the same eight-gene input panel and the same validation framework. Before model fitting, gene expression values in each cohort were preprocessed by log_2_ transformation (where applicable) and standardized to zero mean and unit variance across samples (z-score). The network comprised an input layer with eight features, a single hidden layer, and two softmax output units encoding binary class labels (gastric cancer vs normal tissue). Neuronal activations were computed as weighted sums with bias terms and propagated through a differentiable transfer function (25).

To mitigate overfitting and obtain robust performance estimates in TCGA-STAD, we used repeated 10-fold cross-validation (10 folds × 5 repeats) with embedded hyperparameter tuning. The number of hidden neurons and the L2 weight-decay parameter were jointly optimized, using the area under the receiver operating characteristic curve (AUC; computed with pROC) as the primary objective. The configuration achieving the highest cross-validated AUC was selected as the final architecture. To further quantify and correct for potential optimism, we performed 1,000-iteration bootstrap internal validation under the cross-validated optimal configuration: for each resample, the ANN was refit in the bootstrap sample and evaluated both in-sample and out-of-bag; the mean in-sample minus out-of-bag AUC difference was taken as optimism and subtracted from the apparent AUC to yield an optimism-corrected estimate. For external validation, the final TCGA-trained model was applied without retraining to independent GEO cohorts (GSE54129, GSE26899 and GSE13911) after dataset-specific z-score normalization of the eight genes. In each cohort, we reported AUC together with sensitivity, specificity, positive predictive value (PPV), and negative predictive value (NPV) at the decision threshold determined by the Youden index. To explore clinical utility, an ANN gene score—the model-predicted probability of being tumor—was computed for each TCGA-STAD sample and incorporated into multivariable logistic regression with age, sex, and pathological stage to assess incremental diagnostic value beyond standard clinical covariates. To evaluate reproducibility with respect to random initialization, we performed a seed-stability analysis across 100 independent random seeds while retaining the same five-neuron architecture and validation workflow. Model discrimination was assessed using AUC, sensitivity, and specificity. PPV and NPV, where reported, were interpreted only as cohort-level descriptive metrics because they are prevalence-dependent and cannot be directly extrapolated from case-control datasets to population-level screening settings without calibration to external disease prevalence.

### 2.6 Model interpretability

Given the inherently opaque nature of ANN architectures, we employed the SHAP framework to rigorously quantify the contribution of each feature to model predictions (26). Specifically, we used kernel-based SHAP to decompose, for each sample, the predicted probability of gastric cancer into gene-specific contributions. Within this framework, a Shapley value is assigned to each feature, representing its marginal effect on the predictive outcome; thus, SHAP values describe the behavior of the classifier rather than providing direct estimates of clinical hazard. To assess the robustness of these attributions, the ANN was retrained under five independent random initializations, SHAP profiles were recomputed for each instance, and stability was evaluated using Spearman correlations between the resulting global importance vectors. In addition, we generated 50 bootstrap resamples of the test set and recalculated SHAP-based rankings to examine the impact of sampling variability. Together, this SHAP-based interpretability workflow provides a transparent and statistically robust assessment of how each hub gene contributes to ANN predictions, thereby mitigating the “black-box” limitation of neural network models.

### 2.7 Single-cell RNA sequencing analysis

Single-cell RNA-seq data were processed and analyzed using the Seurat R package (27). The dataset comprised ten tissue specimens obtained from six patients, including three primary gastric cancer specimens, six metastatic specimens, and one adjacent non-tumoral specimen (28). Because the present analysis was designed to characterize cellular heterogeneity and candidate-gene expression within gastric cancer tissues, the primary analysis was restricted to the nine tumor specimens. The single non-tumoral specimen was excluded because it lacked biological replication and therefore could not support a statistically meaningful tumor-versus-non-tumor comparison. Quality control retained cells with mitochondrial gene content <20%, >200 detected genes, and total gene counts between 200 and 6,000, and present in ≥3 cells. Following quality control, raw RNA counts were retained in the unintegrated RNA assay for expression-based downstream analyses. Expression normalization and variance stabilization were performed using SCTransform, with mitochondrial transcript proportion included as a technical covariate. Principal-component analysis was conducted using the SCT assay, and specimen-associated technical variation was addressed using Harmony with sample identity as the batch variable (29). Harmony-corrected dimensions were used for nearest-neighbor graph construction, clustering, and UMAP visualization.

Cell identities were initially assigned using canonical lineage markers and cluster-specific differential-expression signatures. To provide an independent reference-based assessment and reduce reliance on subjective marker interpretation, SingleR was applied to the unintegrated normalized RNA-expression matrix using the Human Primary Cell Atlas (HPCA) reference dataset obtained through the celldex package. SingleR-derived labels were compared with marker-based assignments after harmonization to the same eight major cellular lineages. Clusters showing discordant assignments were re-evaluated using canonical markers and cluster-specific expression profiles, and final cell identities were assigned by consensus. Formal cell-type differential-expression analysis was performed using the Wilcoxon rank-sum test implemented in Seurat. For each major cellular lineage, cells assigned to that lineage were compared with all remaining cells using the unintegrated RNA assay and the FindAllMarkers function, with test.use = “wilcox”, only.pos = FALSE, min.pct = 0.10, and logfc.threshold = 0. Bonferroni-adjusted P values returned by Seurat were used to evaluate statistical support. To investigate potential cellular mechanisms linking candidate hub proteins to gastric cancer progression, we examined their expression distributions across annotated cell types.

### 2.8 Analysis of cell-cell communication

Cell–cell communication was inferred using CellChat v2.1.2 (30). The unintegrated normalized expression matrix from the RNA assay and the final annotations of the eight major cell populations were used as input. Curated human ligand–receptor–cofactor relationships were obtained from the CellChatDB.human database. Overexpressed signaling genes and ligand–receptor interactions were identified using identifyOverExpressedGenes and identifyOverExpressedInteractions, respectively. Communication probabilities were subsequently estimated using computeCommunProb with raw.use = TRUE, such that the inference was based on the normalized, unprojected expression data rather than protein–protein-interaction-network-projected values. CellChat-generated permutation-based P values were used to assess statistical support, and inferred ligand–receptor pairs with P < 0.05 were retained for downstream network summarization. For each sender–receiver cell-type pair, the interaction count was defined as the number of statistically supported inferred ligand–receptor pairs. The interaction weight was defined as the aggregate CellChat-derived communication probability across the statistically supported ligand–receptor pairs connecting the corresponding sender and receiver populations. These quantities were summarized using the aggregated CellChat communication network.

### 2.9 Analysis of pseudo-time series

Pseudotime analysis was performed in Monocle2 on the normalized gene expression matrix as an exploratory approach to order cells along a low-dimensional continuum of transcriptional states. Highly variable genes were used to construct trajectories that summarize gradual changes in gene expression across clusters. Given that the dataset comprises multiple lineages, we interpret these trajectories as abstract axes of transcriptional remodeling within the tumor microenvironment rather than as literal developmental lineages.

### 2.10 Analysis of spatial transcriptome data

Spatial transcriptomic datasets from gastric cancer sections GSM7990473 and GSM7990474 were obtained from GSE251950. Spot-level count matrices and spatial coordinates were processed independently using Seurat v5. Low-quality spots were removed, after which gene-expression data were normalized and variance-stabilized using SCTransform with sequencing depth included as a technical covariate. Cell-type composition at each spatial location was estimated using CARD (31). For each section, a CARD object was constructed using createCARDObject (32), with the annotated single-cell RNA-sequencing dataset used as the reference and the spatial transcriptomic profiles used as the mixture data. Spatial deconvolution was subsequently performed using CARD_deconvolution.

The spatial distributions of the network-prioritized genes were visualized separately in each section. To formally assess whether the observed expression patterns exhibited non-random spatial organization, Global Moran’s I was calculated for each gene using a spatial-neighbor graph derived from the spot coordinates. Moran’s I values greater than zero indicate positive spatial autocorrelation, whereby locations with similar expression values occur closer together than expected under spatial randomness. Statistical significance was evaluated using a randomization-based Moran’s test with the alternative hypothesis of positive spatial autocorrelation. P values were adjusted separately within each section across the candidate genes using the Benjamini–Hochberg procedure, and an adjusted P value <0.05 was considered statistically significant.

### 2.11 Kaplan–Meier plotter database analysis

The association between hub gene mRNA expression and gastric cancer patient prognosis was evaluated using the Kaplan–Meier plotter database (www.kmplot.com). Patients were stratified into high and low-expression cohorts based on median gene expression, and hazard ratios (HRs) with 95% confidence intervals (CIs) and log-rank P values were automatically computed to assess survival differences (33).

### 2.12 Multiplex immunofluorescence staining

This study was conducted in accordance with the Declaration of Helsinki. The use of archival gastric adenocarcinoma tissue specimens for multiplex immunofluorescence staining was reviewed and approved by Ethics Committee of The Affiliated Lianyungang Hospital of Xuzhou Medical University/The First People’s Hospital of Lianyungang, Lianyungang, China (Approval No. KY-20251120003-01). Given the retrospective design and the use of de-identified pathological specimens, the requirement for written informed consent was waived by the committee.

FFPE gastric cancer tissue specimens from n = 5 patients with primary gastric adenocarcinoma who underwent curative gastrectomy were sectioned at 5 µm and mounted on glass slides. For multiplex immunofluorescence, sections were deparaffinized in xylene-free dewaxing solution (G1128, Servicebio) and rehydrated through graded ethanol (100%, 95%, 80% and 70%) to distilled water at room temperature. Antigen retrieval was performed in citrate buffer (pH 6.0, G1202) or EDTA buffer (pH 8.0 or 9.0, G1206/G1203, Servicebio) using a microwave oven according to the antigen requirement. After cooling, endogenous peroxidase was blocked with 3% H_2_O_2_ for 25 min, followed by blocking with 10% normal rabbit serum or 3% BSA for 30 min at room temperature. Sections were then incubated overnight at 4 °C with the first primary antibody diluted in sterile PBS. The next day, slides were washed in PBS and incubated for 50 min with the corresponding HRP-conjugated secondary antibody, followed by incubation with the appropriate TSA fluorophore for 10 min and washing in TBST. Microwave treatment in citrate buffer (pH 6.0) was used to strip non-covalently bound antibodies between staining cycles, and the above procedure was repeated for the second and third primary antibodies. After the final TSA development, nuclei were counterstained with DAPI (G1012, Servicebio), tissue autofluorescence was reduced with an autofluorescence quencher (G1221, Servicebio), and sections were mounted with an anti-fade medium (G1401, Servicebio). Multichannel images were acquired using a Nikon fluorescence microscope and a Pannoramic MIDI scanner (3DHISTECH).

### 2.13 Virtual screening and molecular docking

The candidate small molecules were obtained from the ZINC database, and receptor three-dimensional structures were retrieved from UniProt and the RCSB PDB. ZINC provides commercially available compounds with favorable drug‐like properties and 3D conformations. Ligands were prepared in Schrödinger Maestro 12.8 (LigPrep) by adding hydrogens, adjusting ionization states at pH 7.0 ± 2.0, and energy‐minimizing under the OPLS2005 force field to generate optimized 3D geometries (34). Target proteins—hub proteins implicated in gastric cancer—were identified via UniProt entries, and their PDB structures were downloaded for processing in AutoDock Tools: removal of water molecules, addition of polar hydrogens, Gasteiger charge assignment, and conversion to PDBQT format, with ligands processed analogously to meet AutoDock Vina input requirements. Initial virtual screening of the prepared ZINC compound library was conducted in Maestro’s Virtual Drug Screening module (Schrödinger, accessed May 16, 2025) to exclude unstable or low‐potential candidates. Selected compounds were then docked using AutoDock Vina (35). Docking grids (20 Å × 20 Å × 20 Å) encompassed each protein’s active site, and binding affinities (kcal/mol) were ranked, with lower scores indicating stronger predicted binding; results were visualized as a heatmap of docking scores.

### 2.14 Molecular dynamics simulations

Molecular dynamics simulations were performed in GROMACS 2022.3 (36, 37). Ligand parameters were generated with AmberTools22 under the GAFF force field, and AM1-BCC/RESP charges were assigned following geometry optimization and hydrogen addition in Gaussian 16W. Protein topologies were built using the Amber99SB-ILDN force field. Each complex was solvated in a TIP3P water box, neutralized with Na^+^ ions, and energy-minimized via the steepest-descent algorithm. Equilibration comprised 100 ps each of NVT and NPT ensembles (τ = 0.1 ps, T = 300 K, P = 1 bar). Production runs of 100 ns (5 × 10^6 steps, Δt = 2 fs) were then conducted. Trajectories were analyzed to derive root-mean-square deviation (RMSD), radius of gyration (Rg), and solvent-accessible surface area (SASA). Binding free energies and energy landscapes were calculated using the MM/GBSA method to assess complex stability and energetic profiles (38).

### 2.15 Cell experiments

#### 2.15.1 Materials

The human gastric cancer cell line AGS was obtained from the Shanghai Institute of Biochemistry and Cell Biology (Shanghai, China). RPMI 1640 medium, EdU-555 Cell Proliferation Kit (KGA9606-500) and Annexin V-APC/PI Dual Staining Kit for Apoptosis Detection (KGA1107-20) were purchased from KeyGEN Biotech (Nanjing, China). Fetal bovine serum (FBS) was purchased from Clark Bioscience (Shanghai, China). Trypsin solution and protease inhibitor cocktail were purchased from VICMED (Xuzhou, China). RIPA buffer, Phosphatase Inhibitor Cocktail (PR20015), ECL chemiluminescence kit, anti-GAPDH (10494-1-AP), anti-BAX (50599-2-Ig), anti-BCL2 (12789-1-AP), anti-PI3K (60225-1-Ig), anti-β-Actin (66009-1-Ig), HRP-conjugated Affinipure Goat Anti-Rabbit IgG (H+L) (SA00001-2) were obtained from Proteintech (Wuhan, China). anti-p-PI3K (ab182651) was purchased from Cell Signaling Technology (Cambridge, UK). Caspase-3 (9662S), anti-cleaved Caspase-3 (9661S) were purchased from Abcam (Boston, USA). The BCA Protein Assay Kit, blocking buffer and Cell Counting Kit-8 (CCK-8) reagent were purchased from NCM Biotech (Suzhou, China). PVDF membranes were purchased from Millipore (MA, USA), and a chemiluminescence bioimaging system was obtained from ProteinSimple (SV, USA). Transwell chambers were purchased from Corning (NY, USA), and Matrigel (1:8 dilution) was obtained from BD Biosciences (San Jose, CA, USA). Proflavine hemisulfate (purity > 98%, HY-B0883) and recombinant human TGFB3 (rhTGFB3; purity > 95% by reducing SDS–PAGE, HY-P700152AF) were purchased from MedChemExpress (NJ, USA).

#### 2.15.2 Cell viability assay

To evaluate the growth-inhibitory effect of proflavine hemisulfate (PFH) on AGS cells, cell viability was assessed using a Cell Counting Kit-8 (CCK-8) assay. AGS cells were seeded into 96-well plates at a density of 5 × 10³ cells per well and allowed to adhere overnight. The medium was then replaced with fresh culture medium containing graded concentrations of PFH (1, 2, 4, 8, 16, and 32 μM) and cells were incubated for 24 h at 37 °C in a humidified atmosphere with 5% CO_2_. Subsequently, 10 μL of CCK-8 solution was added to each well and plates were incubated for an additional 30 min under the same conditions. Absorbance at 450 nm was measured using a microplate reader, and cell viability was calculated relative to the untreated control group. The resulting dose–response curve was used to quantify the antiproliferative activity of PFH and to define an effective working concentration range for downstream functional experiments.

#### 2.15.3 EdU proliferation assay

To directly evaluate the antiproliferative effect of proflavine hemisulfate (PFH), 5-ethynyl-2’-deoxyuridine (EdU) incorporation assays were performed. AGS cells were seeded into 24-well plates at a density of 3 × 10^4 cells per well and assigned to four treatment conditions: control, rhTGFB3, PFH, and rhTGFB3 + PFH. After 24 h of treatment, cells were incubated with EdU working solution for 3 h according to the manufacturer’s instructions to label newly synthesized DNA. Nuclei were counterstained with Hoechst to visualize total cell numbers. Images were acquired using a fluorescence microscope under identical acquisition settings, and cell proliferation was quantified as the percentage of EdU-positive (red) cells relative to the total Hoechst-positive (blue) cells.

#### 2.15.4 Colony formation assay

To assess the long-term effects of PFH on AGS cell proliferation and survival, colony formation assays were performed. AGS cells were seeded into 6-well plates at a low density of 1500 cells per well and continuously cultured for 7 days in complete medium containing vehicle (control), rhTGFB3, PFH, the combination of rhTGFB3 and PFH. At the end of the incubation period, colonies were fixed with 4% paraformaldehyde and stained with 1% crystal violet. Colonies containing more than 50 cells were imaged and counted to quantify clonogenic growth.

#### 2.15.5 Transwell invasion assay

To evaluate the inhibitory effect of PFH on the invasive capacity of AGS cells, Transwell invasion assays were performed using Matrigel-coated inserts. AGS cells (5 × 10^4 per insert) were resuspended in serum-free medium containing vehicle (control), rhTGFB3, PFH, or the combination of rhTGFB3 and PFH, and seeded into the upper chambers. The lower chambers were filled with medium supplemented with 10% fetal bovine serum as a chemoattractant. After 24 h of incubation, non-invading cells on the upper surface of the membranes were gently removed with absorbent swabs. Cells that had invaded to the lower surface were fixed and stained with crystal violet, then imaged and counted under a microscope to quantify invasive potential.

#### 2.15.6 Wound healing assay

To examine the effect of PFH on cell motility, a wound-healing assay was performed. AGS cells were seeded into 6-well plates and grown to a confluent monolayer, after which a linear scratch was created across the cell layer using a sterile pipette tip. Detached cells were removed by gentle washing, and the medium was replaced with serum-free medium containing vehicle (control), rhTGFB3, PFH, or the combination of rhTGFB3 and PFH. Phase-contrast images of the wound area were acquired at 0 h and 24 h using a light microscope. Wound closure was quantified in ImageJ by measuring the scratch area at each time point, and the rate of wound healing was calculated to assess lateral migratory capacity.

#### 2.15.7 Cell apoptosis assay

AGS cells were seeded into 24-well plates at a density of 1 × 10^5 cells per well and allowed to adhere. Cells were then treated for 24 h at 37 °C in a humidified incubator with 5% CO_2_ under four conditions: vehicle (control), rhTGFB3, PFH, or the combination of rhTGFB3 and PFH, with three replicate wells per group. After treatment, culture supernatants were removed and cells were washed once with PBS. Cells were detached with 500 μL trypsin solution with EDTA, and trypsinization was stopped by adding 500 μL RPMI-1640 medium supplemented with 10% fetal bovine serum. Cell suspensions were collected by centrifugation at 1,000 rpm, resuspended in 1 mL PBS, and centrifuged again. The resulting pellets were resuspended in 100 μL binding buffer, followed by addition of 5 μL Annexin V–APC and 5 μL propidium iodide (PI). Samples were incubated for 5 min at room temperature in the dark and then immediately analyzed by flow cytometry. Apoptotic cells were quantified based on Annexin V/PI staining profiles.

#### 2.15.8 Western blot analysis

Exponentially growing AGS cells were seeded into 6-well plates and allowed to adhere. Cells were then treated for 24 h with vehicle (control), recombinant human TGF-β3 (rhTGFB3), proflavine hemisulfate (PFH), or the combination of rhTGFB3 and PFH. After treatment, cells were washed twice with ice-cold PBS and lysed on ice in RIPA lysis buffer to extract total cellular protein. Lysates were clarified by centrifugation, and protein concentrations were determined using a BCA assay. Equal amounts of protein were resolved by SDS–PAGE and electrotransferred onto PVDF membranes.

For analysis of apoptosis-related proteins, membranes were blocked with 5% non-fat dry milk in TBST for 1 h at room temperature and incubated overnight at 4 °C with primary antibodies against cleaved Caspase-3, total Caspase-3, BAX and BCL2. After extensive washing in TBST, membranes were incubated with HRP-conjugated secondary antibodies for 1 h at room temperature. Protein bands were visualized using an enhanced chemiluminescence (ECL) substrate and imaged with a chemiluminescence documentation system. β-Actin served as the internal loading control to correct for variations in protein loading, and these blots were used to assess whether PFH promotes apoptotic signaling.

For analysis of PI3K pathway activation, cells were processed in parallel, except that RIPA lysis buffer was supplemented with a phosphatase inhibitor cocktail. After transfer, membranes were blocked with 5% BSA in TBST for 1 h at room temperature, and primary antibodies against phosphorylated PI3K (p-PI3K) and total PI3K were diluted in BSA-containing TBST and incubated overnight at 4 °C. Following TBST washes, membranes were incubated with the appropriate HRP-conjugated secondary antibodies for 1 h at room temperature, and signals were detected by ECL as above. GAPDH was used as the loading control for these blots. Quantification of p-PI3K and PI3K enabled evaluation of TGFB3-induced PI3K activation and determination of whether PFH attenuates this downstream signaling.

### 2.16 NicheNet analysis and in silico TGFBR2 perturbation

To further investigate a potential stromal-to-epithelial route of TGFB3-associated signaling, NicheNet analysis was performed with fibroblasts defined as the sender population and epithelial cells as the receiver population. TGFB3 was specified as the ligand of interest, and candidate epithelial receptors and downstream ligand-responsive genes were prioritized using the curated ligand–receptor and ligand–target prior models implemented in NicheNet. This analysis was used to identify plausible receptor-mediated routes through which fibroblast-derived TGFB3 could be associated with epithelial transcriptional responses. The cell-level association between TGFB3 and the candidate epithelial receptor TGFBR2 was evaluated using Spearman rank-correlation analysis.

To explore the transcriptional consequences potentially associated with disruption of receptor-mediated TGFB3 signaling, an in silico knockout of TGFBR2 was performed in epithelial cells using scTenifoldKnk. The algorithm reconstructed a single-cell gene-regulatory network and compared the original and TGFBR2-perturbed network states to identify genes exhibiting significant manifold displacement after virtual knockout. The significantly perturbed genes were subsequently subjected to functional enrichment analysis.

## 3 Results

### 3.1 MR analysis of circulating plasma proteins associated with GC

**Figure 1:**
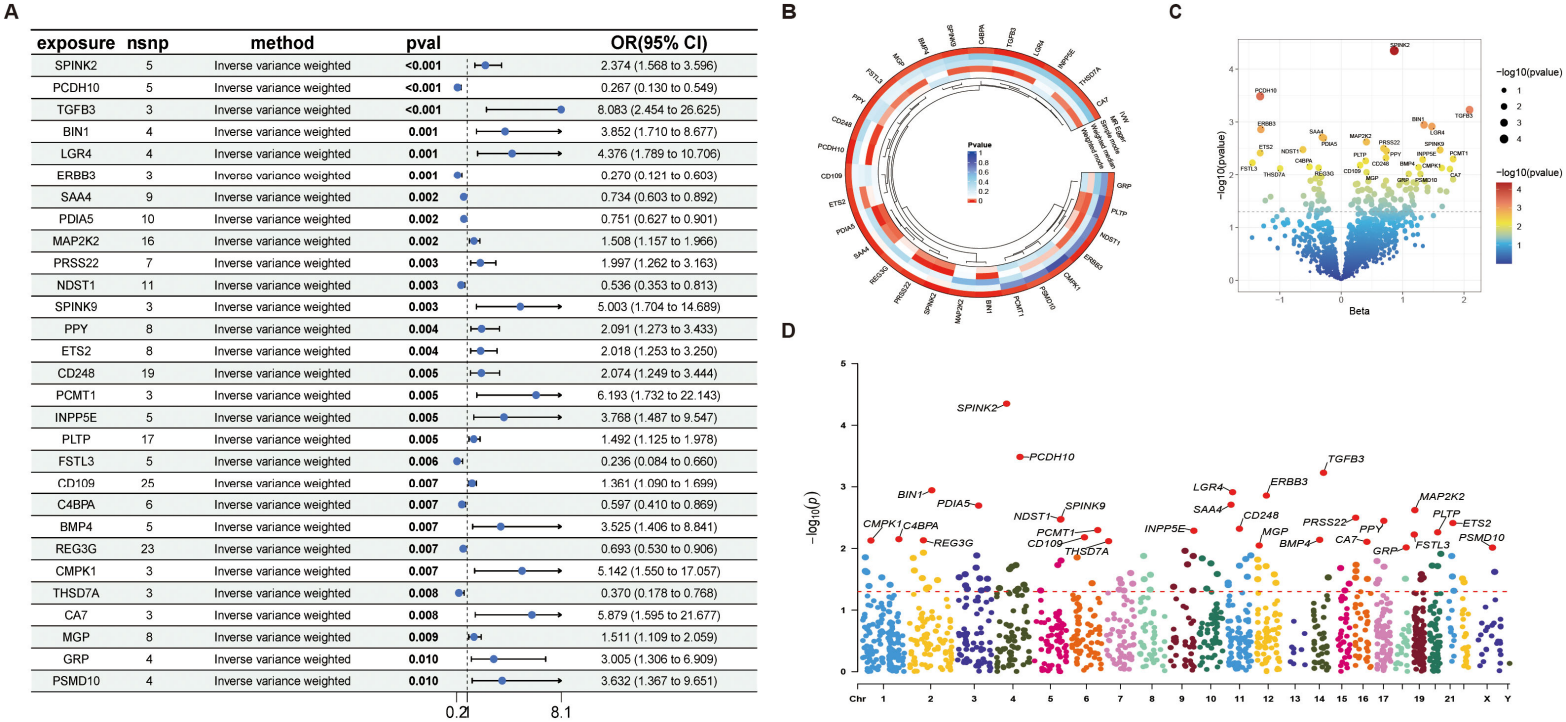
**(A)** Forest plot of odds ratios (ORs) and 95% confidence intervals for 29 plasma proteins causally associated with gastric cancer risk. Estimates were derived using the IVW method; two-sided P values shown are adjusted (P < 0.05). **(B)** Circular heatmap summarizing method-specific MR evidence for the 29 MR-prioritized proteins across five estimators, including IVW, MR-Egger, simple mode, weighted median and weighted mode. Color intensity denotes −log10(P), with warmer colors indicating stronger statistical evidence. **(C)** Volcano plot showing MR beta coefficients on the x-axis and −log10(P) values on the y-axis across the tested plasma proteome. All 29 associated proteins are labeled. **(D)** Manhattan plot showing the genomic distribution of protein–GC MR associations. Each point represents one tested plasma protein locus, and labeled red points indicate the 29 proteins passing threshold.

We first investigated whether genetically predicted circulating plasma protein abundance could be leveraged to prioritize proteins associated with susceptibility to gastric cancer (GC). Using deCODE plasma proteomic pQTLs as exposures and GWAS summary statistics for gastric cancer from the IEU OpenGWAS database (ebi-a-GCST90435577) as the outcome, we performed a proteome-wide two-sample Mendelian randomization (MR) analysis encompassing 4,907 circulating plasma proteins. Among proteins with valid genetic instruments, 29 proteins survived false-discovery-rate (FDR) correction and were retained as the complete MR-prioritized discovery set (**Figure 1A**). These MR-prioritized proteins exhibited bidirectional associations with GC risk, indicating that genetically predicted perturbations in the circulating proteome capture both risk-enhancing and risk-reducing signals. Increased genetically predicted abundance of PCDH10, ERBB3, SAA4, PDIA5, NDST1, FSTL3, C4BPA, REG3G, and THSD7A was associated with a reduced risk of GC. Conversely, increased genetically predicted abundance of SPINK2, TGFB3, BIN1, LGR4, MAP2K2, PRSS22, SPINK9, PPY, ETS2, CD248, PCMT1, INPP5E, PLTP, CD109, BMP4, CMPK1, CA7, MGP, GRP, and PSMD10 was associated with an elevated risk of GC. Multi-method MR analyses further provided complementary evidence for the robustness of several associations across IVW, MR-Egger, simple mode, weighted median, and weighted mode estimators (**Figure 1B**). Viewed across the proteome-wide distribution of MR effect estimates and statistical evidence, these prioritized proteins were not confined to a single genomic locus or pathway class. Rather, they represented a genome-wide repertoire of candidate circulating protein signals genetically linked to GC susceptibility (**Figures 1C, D**).

To evaluate the robustness and generalizability of the protein–disease relationships, we next repeated the MR analyses in two independent gastric cancer GWAS datasets. In a larger European GWAS (ebi-a-GCST90018849; 1,029 cases and 290,685 controls), eight proteins reproduced the directions of effect observed in the discovery analysis: genetically higher ERBB3 levels were consistently associated with lower gastric cancer risk, whereas elevated TGFB3, CD248, GRP, LGR4, ETS2, MGP and BMP4 conferred higher risk (**Supplementary Table 5**). In the East-Asian BioBank Japan GWAS (bbj-a-119; 6,563 cases and 195,745 controls), we again observed fully concordant effect directions despite differences in ancestry and LD structure (**Supplementary Table 6**).

To further validate the robustness of the 29 plasma proteins identified by MR as causally associated with gastric cancer risk, we performed formal tests for horizontal pleiotropy and heterogeneity for each protein. The MR–Egger intercept was used to detect directional pleiotropy, that is, whether genetic instruments influence gastric cancer risk through pathways other than the target protein. All 29 proteins exhibited MR–Egger intercept P values > 0.05, indicating no significant horizontal pleiotropy and thereby reducing concern about major instrument-related bias in the genetic instruments (**Supplementary Table 7**). Heterogeneity across single-nucleotide polymorphism (SNP) instruments was assessed using Cochran’s Q statistic. Again, all proteins yielded Q-test P values > 0.05, demonstrating no significant inconsistency among instrument-specific effect estimates (**Supplementary Table 8**). MR-PRESSO global tests did not detect substantial horizontal pleiotropy after correction for multiple testing, and did not identify outlier variants among proteins for which the test was estimable. Leave-one-out analyses confirmed that no single SNP drove the observed associations (**Supplementary Table 9**). Finally, cis-only and trans-only MR analyses yielded effect directions generally concordant with the combined cis+trans models and, for most proteins, retained statistical significance in the cis-only setting (**Supplementary Tables 10**, **11**), indicating that the main conclusions are not dependent on a particular class of instruments.

### 3.2 Functional annotation and pathway enrichment analysis

**Figure 2:**
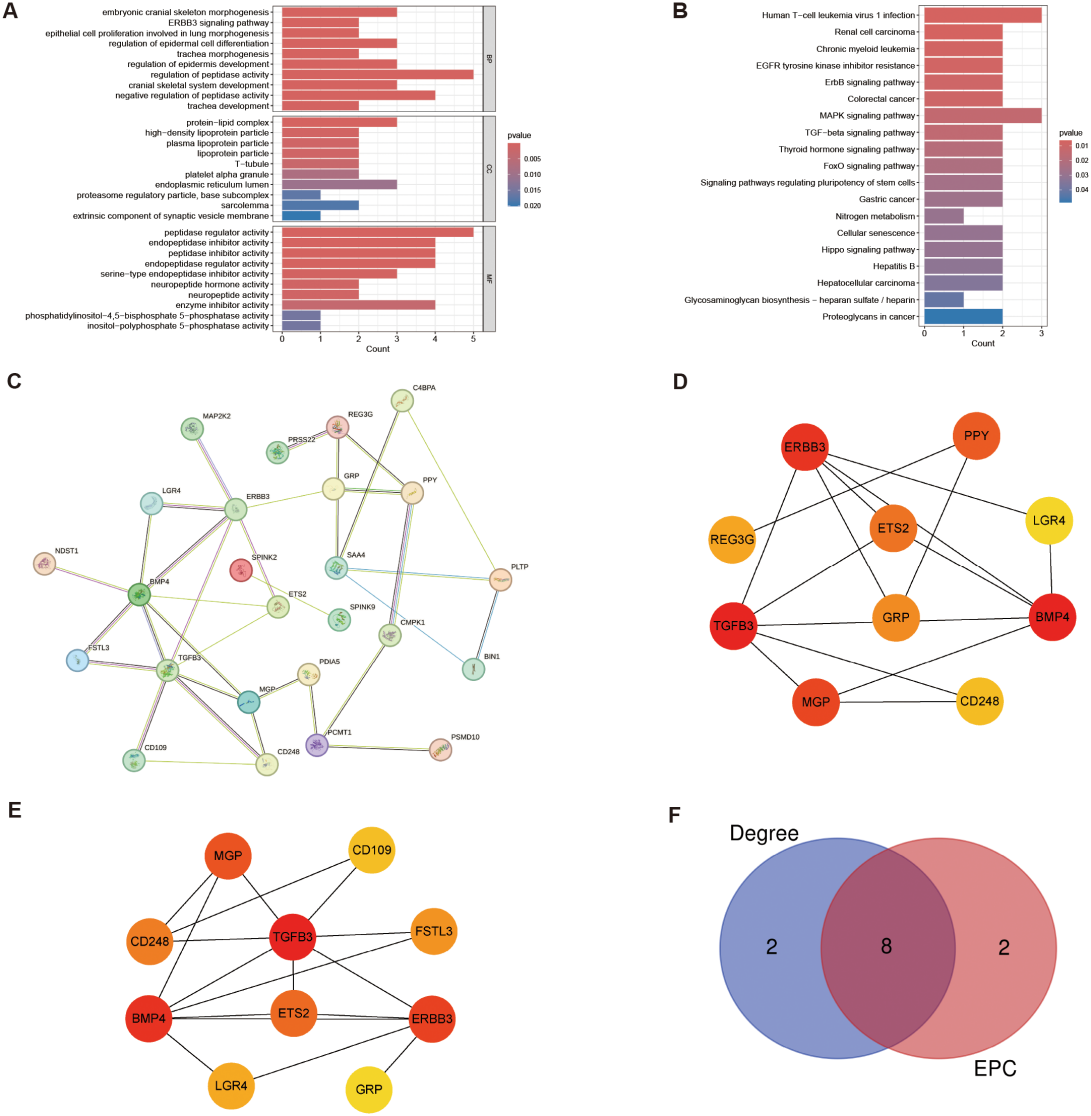
**(A)** Results of GO enrichment analysis. **(B)** Results of KEGG pathway enrichment analysis. **(C)** Protein–protein interaction network of 29 circulating plasma proteins significantly associated with gastric cancer. **(D)** Top ten nodes ranked by degree centrality. **(E)** Top ten nodes ranked by Edge Percolated Component (EPC). **(F)** Venn diagram of network-prioritized genes.

We next performed exploratory functional annotation to place the 29 MR-prioritized plasma proteins within a biological framework. Gene Ontology analysis revealed that the dominant biological processes were associated with epithelial growth and differentiation, ErbB-related signaling, and regulation of protease activity (**Figure 2A**), pointing to coordinated programs involved in epithelial-state control and stromal remodeling. Cellular component and molecular function terms were enriched for extracellular and secretory features, lipoprotein-related activity, and protease regulatory functions, consistent with the circulating and microenvironment-associated properties of these candidates. KEGG analysis further linked these proteins to canonical cancer pathways, including ErbB, MAPK, and TGF-β signaling, together with EGFR tyrosine kinase inhibitor resistance and gastric cancer-related pathways (**Figure 2B**). Together, these results suggest that MR-prioritized plasma proteins may contribute to gastric cancer biology by modulating epithelial development, lipid-associated processes, protease activity, and oncogenic signaling. This functional landscape provided the rationale for subsequent protein-level and multi-omic characterization.

### 3.3 Exploratory PPI network construction and network-based candidate prioritization

We next used STRING to construct an exploratory PPI network comprising the 29 plasma proteins genetically prioritized for gastric cancer risk (**Figure 2C**). The network was imported into Cytoscape and interrogated using cytoHubba. Because PPI databases are susceptible to annotation and literature-density biases, and because topological centrality does not by itself establish causality or functional indispensability, this analysis was interpreted strictly as a hypothesis-generating prioritization framework. Nodes were ranked using two complementary metrics, degree centrality and edge percolated component (EPC), and visualized according to connectivity strength. The top ten proteins ranked by degree centrality (**Figure 2D**) and EPC score (**Figure 2E**) were compared (39), and their intersection was used to minimize dependence on a single centrality definition. This strategy identified eight topology-prioritized candidates—BMP4, CD248, GRP, LGR4, MGP, TGFB3, ERBB3 and ETS2—for subsequent biological interpretation and multi-omic contextualization (**Figure 2F**). We therefore refer to these proteins as network-prioritized candidates.

### 3.4 Construction and validation of the GC ANN predictor

**Figure 3:**
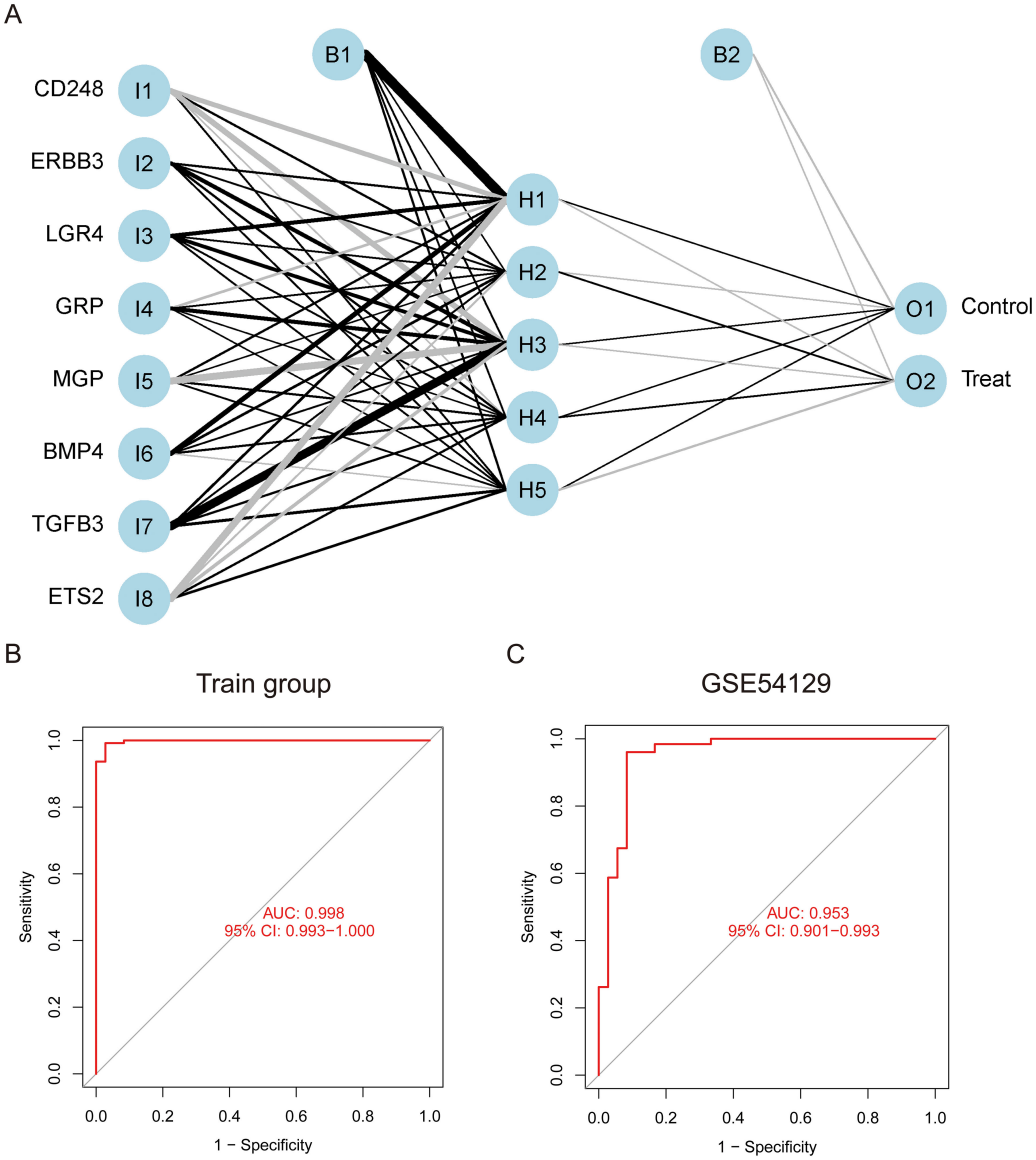
**(A)** Schematic of the multilayer perceptron with eight input nodes (BMP4, CD248, GRP, LGR4, MGP, TGFB3, ERBB3, ETS2), a five-node hidden layer and a two-node output layer encoding Control (normal) and Treatment (GC). **(B)** Receiver operating characteristic (ROC) curve and area under the curve (AUC) in the training cohort (TCGA-STAD). **(C)** ROC curve and AUC in the independent validation cohort (GSE54129).

We developed a multilayer perceptron to classify gastric cancer (GC) versus normal tissue using eight network-prioritized genes—BMP4, CD248, GRP, LGR4, MGP, TGFB3, ERBB3, and ETS2—as inputs. The network comprised an 8-unit input layer, a single hidden layer, and a two-unit softmax output encoding GC and normal classes (**Figure 3A**). Among candidate configurations, a hidden layer with five neurons provided the best trade-off between model complexity and discrimination. In the TCGA-STAD training cohort, the naïve apparent AUC of this network was 0.998 (95% CI, 0.993–1.000; **Figure 3B**), consistent with excellent separation of tumor and non-tumor samples. Because single-fit performance metrics can be overly optimistic, we conducted formal internal validation. Using repeated 10-fold cross-validation with L2 weight decay, the ANN achieved a TCGA cross-validated AUC of 0.913. Bootstrap internal validation with 1,000 resamples yielded an optimism-corrected AUC of 0.908, suggesting that the model retained strong discrimination after correction for potential overfitting. When the locked TCGA-trained model was applied to independent GEO cohorts without retraining, it achieved external AUCs of 0.953 in GSE54129, 0.837 in GSE26899, and 0.819 in GSE13911 (**Figure 3C**; **Supplementary Table 12**). In TCGA-STAD, the corresponding sensitivity and specificity were 0.701 and 0.719, respectively. PPV and NPV were reported as cohort-level descriptive metrics only, given their dependence on disease prevalence and the case-control nature of the analyzed datasets.

To determine whether the ANN provided added performance beyond simpler classifiers, we compared it with logistic regression, ridge logistic regression, LASSO logistic regression, and random forest using the same eight-gene feature set and validation framework (**Supplementary Table 20**). The ANN achieved the strongest overall performance across internal and external validation metrics. Logistic regression achieved a TCGA cross-validated AUC of 0.742 and a bootstrap-corrected AUC of 0.625, with external AUCs of 0.795, 0.673, and 0.755 in GSE54129, GSE26899, and GSE13911, respectively. LASSO logistic regression showed stronger performance than unpenalized logistic regression in TCGA cross-validation and in two external cohorts, but its bootstrap-corrected AUC and GSE54129 AUC remained lower than those of the ANN. Ridge logistic regression and random forest showed weaker or less consistent performance across cohorts. These comparisons suggest that the ANN provided the strongest discrimination under the present validation framework, while the performance of LASSO also supports the robustness of the eight-gene diagnostic signal rather than proving exclusive superiority of the ANN architecture.

To evaluate whether the ANN result depended on a favorable random initialization, we performed a seed-stability analysis across 100 independent random seeds while retaining the same five-neuron architecture and validation workflow (**Supplementary Table 21**). The ANN showed stable central performance across most random initializations, with median TCGA cross-validated AUC of 0.910 (IQR, 0.899–0.922) and median bootstrap-corrected AUC of 0.905 (IQR, 0.894–0.914). External validation performance was also consistent in its central tendency, with median AUCs of 0.951 in GSE54129, 0.834 in GSE26899, and 0.819 in GSE13911. The final locked ANN model used in the main analysis showed performance close to these median values, indicating that it was representative of typical model behavior rather than an unusually favorable initialization. Nevertheless, a small number of seeds yielded substantially lower AUCs, indicating occasional sensitivity to random initialization and stochastic optimization. We therefore interpreted the ANN as a cross-validated and externally validated classifier whose central performance was robust, while acknowledging initialization sensitivity as a reproducibility limitation.

To assess clinical utility, we derived a continuous ANN gene score for each TCGA-STAD sample, defined as the model-predicted probability of being tumor, and incorporated it into multivariable logistic regression alongside age, sex and pathological stage. A clinical-only model including age, sex and stage showed modest discrimination (AUC ≈0.57). Addition of the ANN gene score increased the AUC to ~0.79, with a statistically significant improvement by DeLong’s test (P<0.05). In this multivariable framework, the ANN score remained an independent and strong predictor of tumor status (odds ratio ~6.9 per unit increase; 95% CI ~3.9–9.6), whereas age, sex and stage did not show robust associations in this case–control setting (**Supplementary Tables 13**, **14**). These results suggest that the eight-gene ANN captures molecular information that is not fully represented by the available clinical variables, although prospective and population-calibrated validation will be required before clinical implementation.

### 3.5 SHAP-based interpretability of the GC classifier

**Figure 4:**
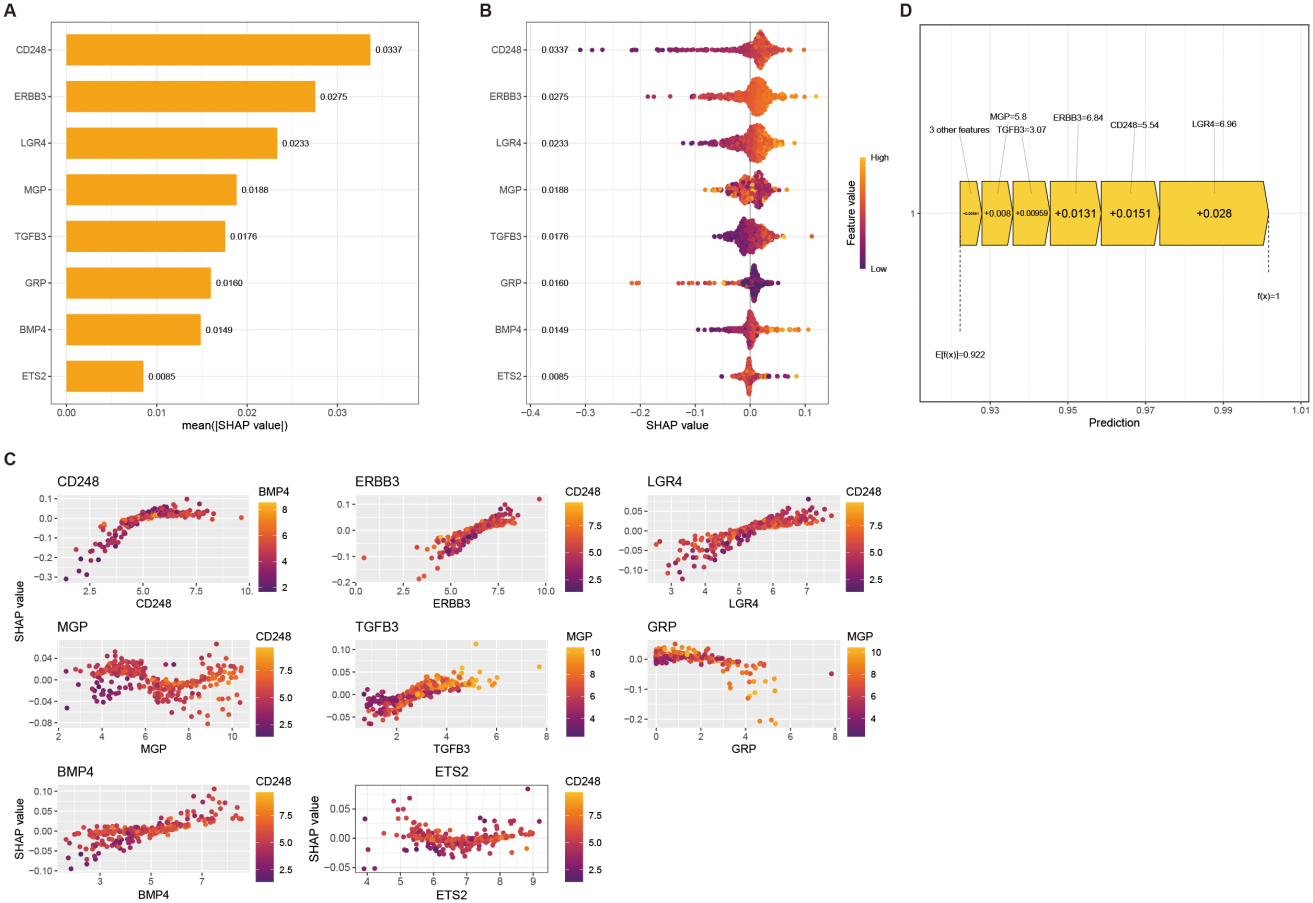
**(A)** ranks genes by global importance (mean absolute SHAP), with bar height quantifying each gene’s contribution to model predictions. **(B)** shows a SHAP beeswarm (summary) plot: each point represents a sample-level contribution for a given gene, horizontally positioned by its SHAP value, vertically jittered to reveal density, and colored by the corresponding feature (expression) level. **(C)** presents SHAP dependence plots for representative genes, plotting SHAP values against normalized expression (or an indicated interacting feature) to resolve nonlinear and interaction-dependent effects. **(D)** summarizes signed contributions to the model output, where positive values indicate an increase in the predicted probability of GC.

To quantify feature contributions at both global and individual levels, we computed SHAP on the trained model. Globally, the ranking by mean absolute SHAP highlighted CD248 as the most influential gene, followed by ERBB3, LGR4, MGP, TGFB3, GRP, BMP4 and ETS2 (mean |SHAP| ≈ 0.0337, 0.0275, 0.0233, 0.0188, 0.0176, 0.0160, 0.0149, and 0.0085, respectively), delineating a clear hierarchy of predictive importance across the eight-gene panel (**Figure 4A**). Consistent with heterogeneous gene–phenotype coupling, SHAP summary (beeswarm) plots revealed broad within-gene dispersion spanning positive and negative contributions, indicating that each marker can either increase or decrease the predicted GC probability depending on its expression context. This heterogeneity underscores the presence of nonlinearity and sample-specific effects that are not captured by simple marginal associations (**Figure 4B**). Dependence plots further resolved these patterns, showing threshold-like and plateau behaviors as well as interaction-modulated effects—for example, the influence of ERBB3 and LGR4 varying with CD248, and the effects of TGFB3 and GRP modulated by MGP. These observations indicate that classifier decisions reflect coordinated, nonlinear and interaction-dependent contributions rather than uniform monotonic effects of individual genes (**Figure 4C**). At the individual level, force plots demonstrated how cumulative gene contributions displace the baseline prediction toward a GC call; in a representative sample, the baseline expectation (E[f(x)] ≈ 0.922) was pushed to a near-certain GC prediction by multiple positive SHAP contributions of modest magnitude, illustrating multi-gene synergy at the decision boundary (**Figure 4D**). Importantly, these SHAP values quantify feature contributions within the ANN classifier and are not intended as direct estimates of clinical or causal risk; the association between gene expression and overall survival was instead evaluated using dedicated Cox models in the prognostic analyses.

To assess robustness to stochastic variation, we trained the ANN under five independent random seeds. Test-set AUCs for these refits ranged from 0.51 to 0.87, with four of five models achieving AUCs between 0.83 and 0.87 (median AUC ≈ 0.85), indicating that high discriminative performance is preserved for most initializations despite occasional unstable fits (**Supplementary Table 15**). Across the same refits, we observed broadly similar global SHAP profiles (pairwise Spearman ρ = 0.70–1.00; median ρ = 0.85; **Supplementary Figure 1A**), with CD248, ERBB3 and LGR4 consistently ranked among the top contributors (**Supplementary Table 16**). Bootstrap resampling of the test set further showed that gene-wise mean |SHAP| profiles were stable with respect to sampling variability, with a median Spearman correlation of 1.00 and a minimum correlation of 0.93 between each bootstrap replicate and the overall mean profile (**Supplementary Figure 1B**). These results indicate that the qualitative hierarchy of ANN feature contributions was reproducible across model refitting and sampling perturbation.

### 3.6 Cell type–specific expression in gastric tumor tissues

**Figure 5:**
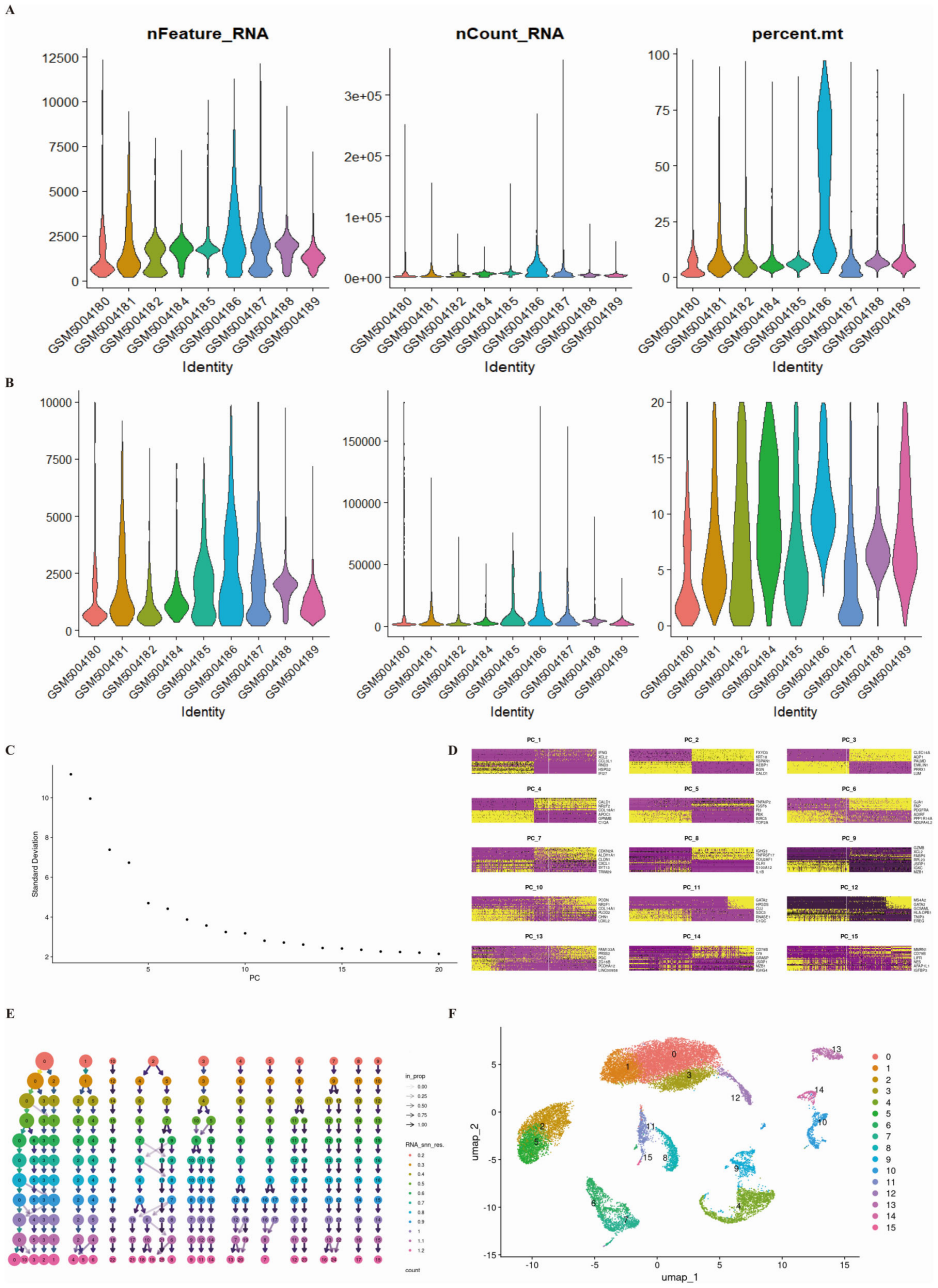
**(A)** Violin plots of detected genes per cell (nFeature_RNA), UMI counts per cell (nCount_RNA) and mitochondrial read fraction (percent.mt) across nine gastric cancer specimens before quality filtering. **(B)** Corresponding violin plots after application of quality‐control thresholds (200 ≤ nFeature_RNA ≤ 6,000; percent.mt ≤ 20%). **(C)** Elbow plot of PC standard deviations for PCs 1–20. **(D)** Heatmaps of PC loadings before and after Harmony integration. **(E)** Hierarchical clustering dendrogram based on the Harmony‐corrected PCs. **(F)** UMAP delineating 16 discrete cell clusters.

The single-cell analysis included nine gastric cancer specimens—three primary tumors and six metastatic lesions—from a six-patient cohort. The only adjacent non-tumoral specimen in GSE163558 was excluded from the tumor-focused analysis because it lacked biological replication for formal tumor-versus-non-tumor comparison. After quality control, 20,491 high-quality cells were retained (**Figures 5A, B**). The first ten principal components were selected for downstream analysis on the basis of the elbow plot (**Figure 5C**). Harmony correction reduced specimen-associated separation in the low-dimensional representation, after which graph-based clustering identified 16 transcriptional clusters (**Figures 5D–F**).

**Figure 6:**
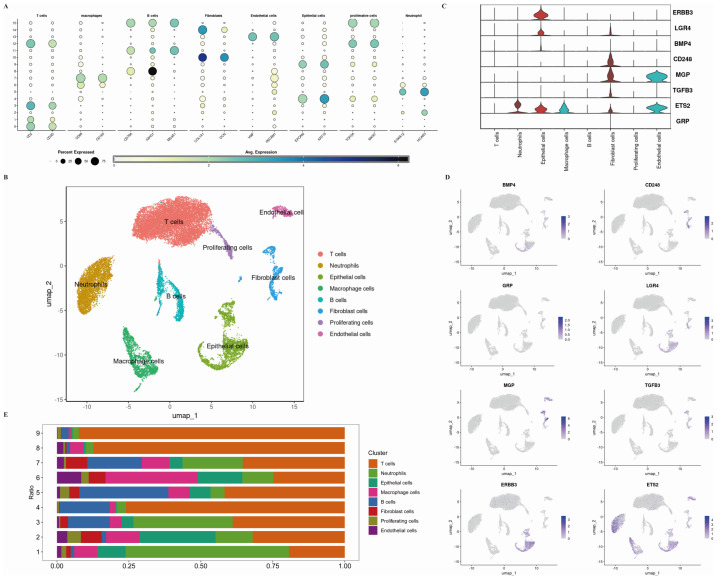
**(A)** Marker genes used for cell‐type annotation. **(B)** Eight cell clusters identified and annotated by canonical marker genes. **(C, D)** Expression distributions of the eight network-prioritized genes across the annotated cell types. **(E)** Bar graph depicting the relative abundance of major cell populations across individual samples.

Following dimensionality reduction and clustering, eight cellular subpopulations were annotated as T cells, neutrophils, epithelial cells, macrophages, B cells, fibroblasts, proliferative cells and endothelial cells (**Figures 6B, E**). Annotation was based on established marker gene expression (**Figure 6A**) (28): T cells (CD2, CD3D), neutrophils (S100A12, HCAR3), epithelial cells (EPCAM, KRT19), macrophages (CD68, CD163), B cells (CD79A, MS4A1, IGHG1), fibroblasts (DCN, COL1A1), proliferative cells (MKI67, TOP2A) and endothelial cells (VWF, PECAM1). To independently evaluate these marker-based assignments, we applied SingleR to the unintegrated normalized RNA-expression matrix. After harmonization of annotation terminology, SingleR-derived labels were highly concordant with the marker-based assignments at the level of the eight major lineages across the 20,491 shared cell labels (**Supplementary Figures 3A, B**). Single‐cell expression profiling of the eight network-prioritized genes (**Figures 6C, D**; **Supplementary Figure 3C**) revealed distinct cell‐type–specific patterns: ERBB3 was markedly upregulated in epithelial cells; LGR4 exhibited moderate expression in epithelial cells and low expression in fibroblasts; BMP4 showed low‐level expression in epithelial cells; CD248 was highly expressed in fibroblasts; MGP was enriched in both fibroblasts and endothelial cells; TGFB3 displayed high expression in fibroblasts; ETS2 was abundantly expressed in neutrophils, epithelial cells, macrophages and endothelial cells, with minimal expression in fibroblasts; and GRP was detected at very low levels in fibroblasts. These findings provide formal cell-level statistical support for lineage-associated expression patterns.

### 3.7 Cell–cell communication and pseudotime trajectory analysis

**Figure 7:**
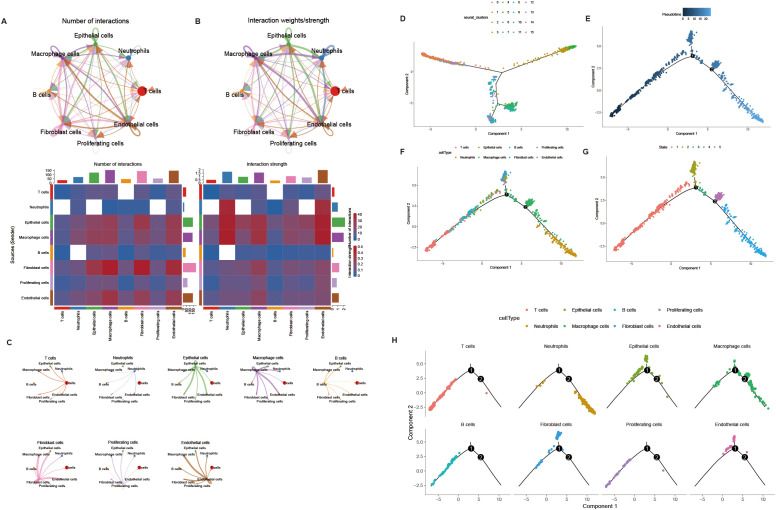
**(A)** CellChat network and heatmap summarizing the number of statistically supported inferred ligand–receptor pairs among the eight annotated cell types. **(B)** CellChat network and heatmap summarizing the aggregate model-derived communication probabilities among the eight annotated cell types. **(C)** Quantitative summary of inferred interaction counts and aggregate communication weights for each sender–receiver cell-type pair. **(D)** Monocle 2 trajectory displaying the distribution of the 16 transcriptional clusters on the inferred low-dimensional manifold. **(E)** The same trajectory colored according to the numerical pseudotime value. **(F)** Distribution of the eight annotated cell types across the inferred trajectory. **(G)** Trajectory colored according to the Monocle-defined state assignments. **(H)** Separate visualization of the eight cell types on the common inferred transcriptional-state manifold.

CellChat analysis identified multiple statistically supported, expression-based ligand–receptor relationships among the eight annotated cellular populations (**Figures 7A–C**). A relatively large number of significant inferred ligand–receptor pairs and higher aggregate CellChat-derived communication probabilities were observed in networks involving macrophages and epithelial cells, fibroblasts and epithelial cells, macrophages and T cells, and fibroblasts and endothelial cells (40). The interaction-count network summarized the number of statistically supported ligand–receptor pairs, whereas the interaction-weight network represented their aggregated model-derived communication probabilities.

Monocle 2 was subsequently used to position the eight major cellular populations along an exploratory transcriptional-state continuum (**Figures 7D–H**). Pseudotime distributions differed substantially among cell types (Kruskal–Wallis χ² = 15,514.71, df = 7, P < 2.2 × 10^-^¹^6^; epsilon-squared = 0.757; **Supplementary Table 22**). In the numerical orientation of the inferred axis, neutrophils showed the lowest median pseudotime value (median = 1.12), followed by macrophages (median = 5.66). B cells and proliferating cells occupied intermediate positions, with median pseudotime values of 16.05 and 18.31, respectively. T cells, endothelial cells, epithelial cells, and fibroblasts were concentrated toward higher numerical pseudotime values, with respective medians of 20.67, 21.44, 21.70, and 21.90. These non-uniform distributions indicate substantial differences in transcriptional-state composition among the annotated populations. However, because the trajectory incorporated biologically distinct lineages and its orientation was not anchored to an independently defined biological starting state, the observed ordering does not imply that these cell types arise from one another or represent sequential stages of tumor progression.

Within-cell-type Spearman analyses further identified statistically supported associations between candidate-gene expression and the inferred pseudotime axis (**Supplementary Table 23**). The strongest positive associations included CD248 in fibroblasts (ρ = 0.587, BH-adjusted P = 9.20 × 10^-8^²), LGR4 in fibroblasts (ρ = 0.475, BH-adjusted P = 5.24 × 10^-50^), LGR4 in epithelial cells (ρ = 0.395, BH-adjusted P = 4.47 × 10^-89^), and ERBB3 in epithelial cells (ρ = 0.317, BH-adjusted P = 1.76 × 10^-56^). ETS2 showed negative associations with pseudotime in macrophages (ρ = −0.547, BH-adjusted P = 2.83 × 10^-^¹³^4^) and neutrophils (ρ = −0.320, BH-adjusted P = 1.64 × 10^-84^). In endothelial cells, positive associations were observed for MGP (ρ = 0.234, BH-adjusted P = 2.07 × 10^-6^), ETS2 (ρ = 0.208, BH-adjusted P = 1.91 × 10^-5^), BMP4 (ρ = 0.140, BH-adjusted P = 0.0054), and TGFB3 (ρ = 0.136, BH-adjusted P = 0.0054).

### 3.8 Spatial transcriptomic analysis of hub gene expression within gastric cancer

**Figure 8:**
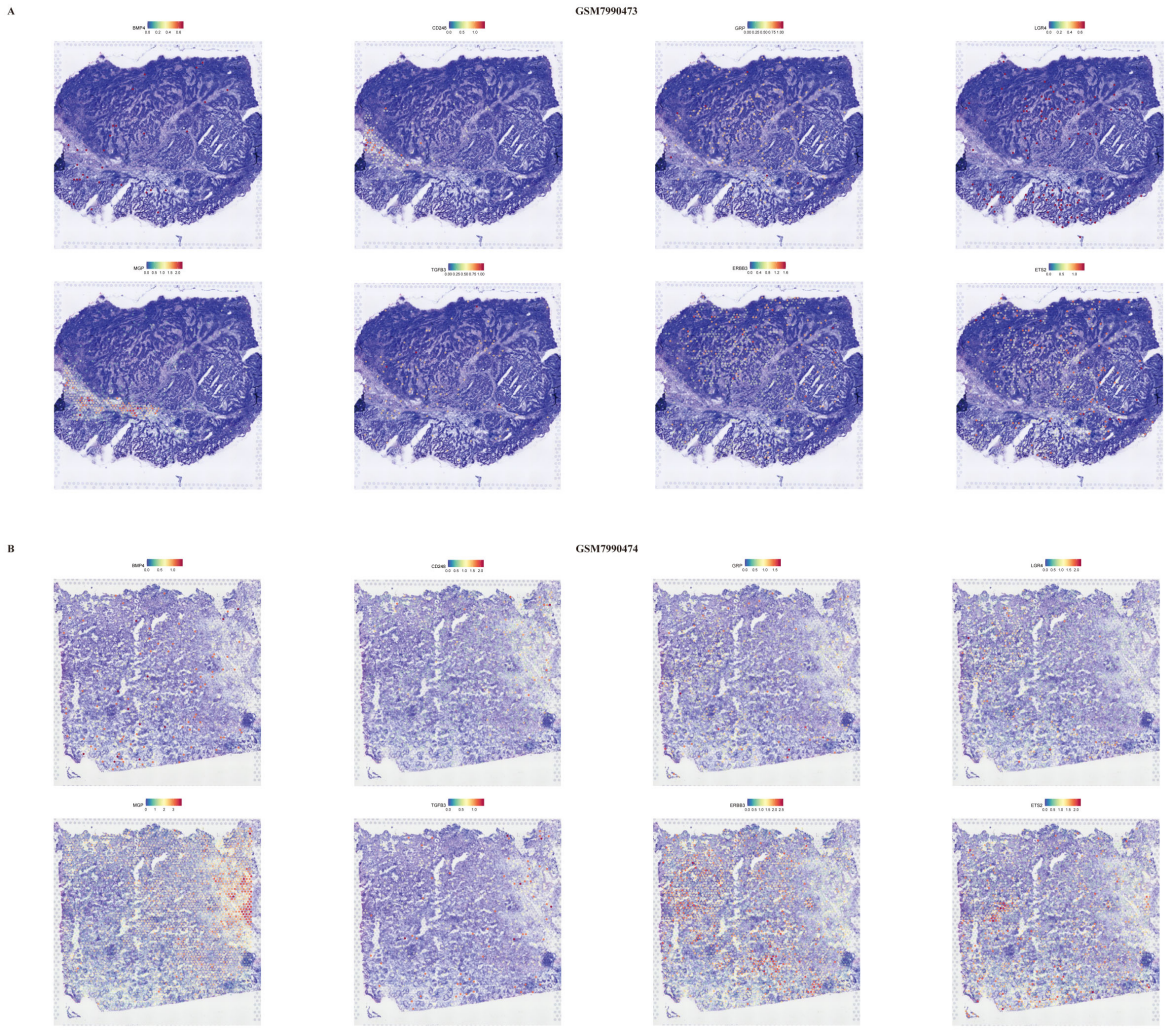
**(A)** Spatial distribution of the eight network-prioritized genes in gastric cancer tissue section GSM7990473. Regions exhibiting more intense red coloration correspond to higher expression levels of the mapped genes. **(B)** Spatial distribution of the eight network-prioritized genes in gastric cancer tissue section GSM7990474. Deeper red hues denote elevated local gene expression.

Spatial visualization of the eight network-prioritized genes revealed heterogeneous expression distributions across the two gastric cancer sections (**Figures 8A, B**). To determine whether these patterns represented statistically supported spatial organization, Global Moran’s I analysis was performed separately for each gene and each section.

All eight genes exhibited statistically significant positive spatial autocorrelation in GSM7990473 (**Supplementary Figure 4A**). MGP showed the largest Moran’s I value (I = 0.289, FDR <0.001), followed by ETS2 (I = 0.228, FDR <0.001), ERBB3 (I = 0.213, FDR <0.001), GRP (I = 0.208, FDR <0.001), LGR4 (I = 0.161, FDR <0.001), and CD248 (I = 0.136, FDR <0.001). TGFB3 and BMP4 also showed statistically significant but comparatively weak positive spatial autocorrelation (TGFB3: I = 0.023, FDR = 0.012; BMP4: I = 0.013, FDR = 0.005). Significant positive spatial autocorrelation was also observed for all eight genes in GSM7990474 (**Supplementary Figure 4B**). MGP again showed the largest effect (I = 0.395, FDR <0.001), followed by ERBB3 (I = 0.197, FDR <0.001), LGR4 (I = 0.120, FDR <0.001), ETS2 (I = 0.107, FDR <0.001), and CD248 (I = 0.093, FDR <0.001). Positive but smaller spatial effects were observed for TGFB3 (I = 0.034, FDR <0.001), GRP (I = 0.032, FDR <0.001), and BMP4 (I = 0.016, FDR = 0.029).

These results demonstrate that the expression of all eight candidate genes was spatially non-random within both analyzed sections, although the magnitude of spatial autocorrelation differed substantially among genes. MGP showed the most consistent spatial clustering across both sections, whereas TGFB3, GRP, and BMP4 generally exhibited weaker spatial autocorrelation.

### 3.9 Survival analysis of GC network-prioritized genes

**Figure 9:**
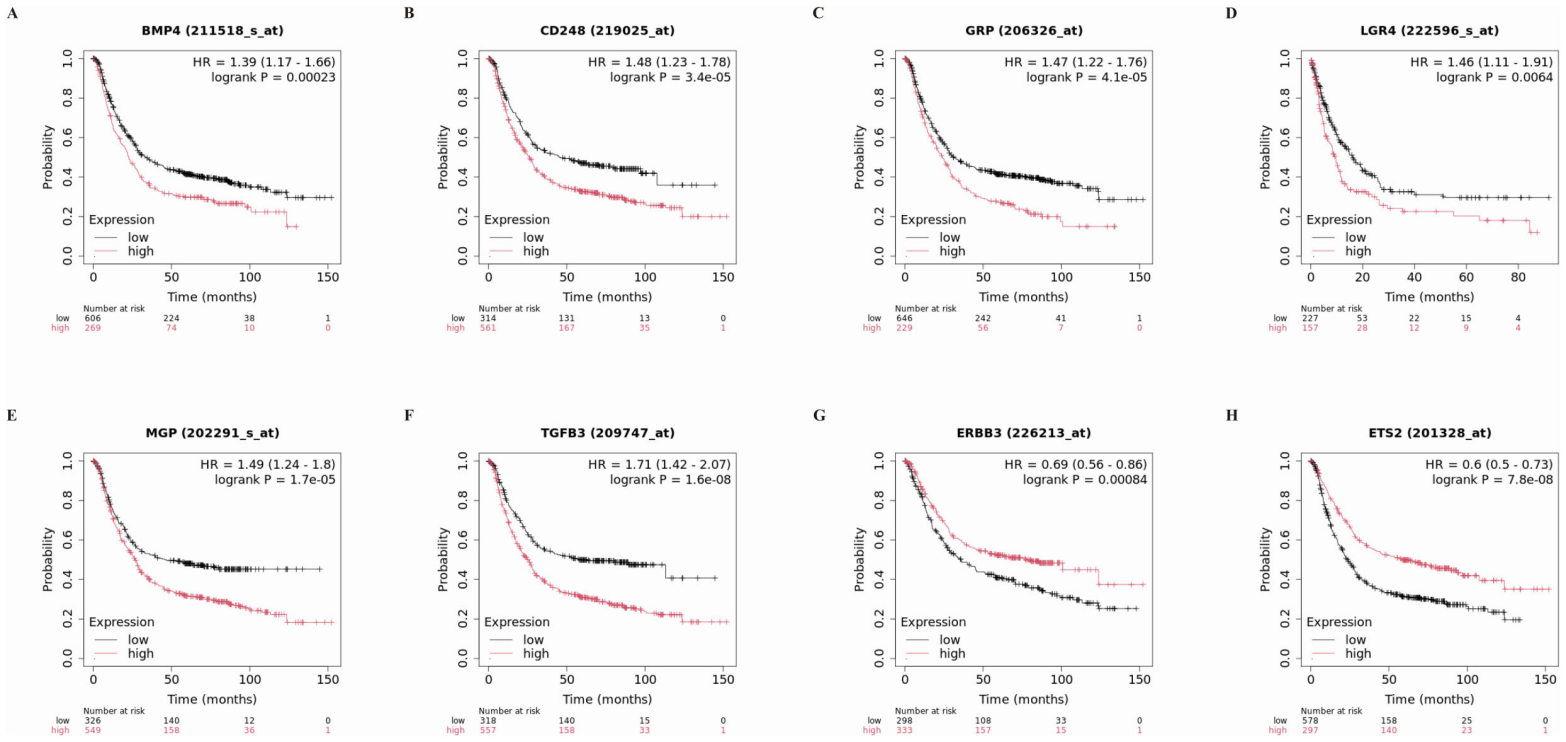
Kaplan–Meier analysis of OS in gastric cancer patients stratified by mRNA expression of network-prioritized genes: **(A)** BMP4, **(B)** CD248, **(C)** GRP, **(D)** LGR4, **(E)** MGP, **(F)** TGFB3, **(G)** ERBB3 and **(H)** ETS2.

Kaplan–Meier Plotter is an online tool for survival analysis based on hub gene expression. GC patients were stratified into high- and low-expression cohorts by the median mRNA level of each hub gene. High expression of BMP4 (HR = 1.39; 95% CI, 1.17–1.66; log-rank P = 2.3 × 10^-4^), CD248 (HR = 1.48; 95% CI, 1.23–1.78; P = 3.4 × 10^-5^), GRP (HR = 1.47; 95% CI, 1.22–1.76; P = 4.1 × 10^-5^), LGR4 (HR = 1.46; 95% CI, 1.11–1.91; P = 6.4 × 10^-^³), MGP (HR = 1.49; 95% CI, 1.24–1.80; P = 1.7 × 10^-5^) and TGFB3 (HR = 1.71; 95% CI, 1.42–2.07; P = 1.6 × 10^-8^) was significantly associated with reduced overall survival (**Figures 9A–F**). In contrast, elevated ERBB3 (HR = 0.69; 95% CI, 0.56–0.86; P = 8.4 × 10^-4^) and ETS2 (HR = 0.60; 95% CI, 0.50–0.73; P = 7.8 × 10^-8^) expression correlated with improved overall survival (**Figures 9G, H**). These findings indicate that BMP4, CD248, GRP, LGR4, MGP and TGFB3 may serve as adverse prognostic biomarkers in gastric cancer, whereas ERBB3 and ETS2 may act as protective prognostic indicators. We then asked how these survival associations relate to the ANN explanations in TCGA-STAD. To this end, we fitted single-gene Cox models for each of the eight MR-prioritized genes and compared the resulting log hazard ratios with their global SHAP importance (mean |SHAP|) derived from the ANN (**Supplementary Figure 2A**). Overall, there was no strong monotonic correlation between Cox coefficients and SHAP importance (Spearman ρ ≈ 0.02, P>0.05; **Supplementary Table 17**), indicating that features exerting the largest marginal effects on overall survival are not necessarily those that contribute most strongly to the classifier’s decision. Nevertheless, canonical high-risk genes such as TGFB3, CD248 and BMP4 displayed both adverse hazard ratios and appreciable SHAP contributions, whereas ERBB3 and ETS2 showed protective hazards yet retained non-negligible Shapley values. These patterns underscore that SHAP captures the functional contribution of each gene to the ANN’s diagnostic boundary, while survival models quantify downstream prognostic effects in a different statistical and biological context.

Finally, to test whether the ANN itself encodes prognostic information beyond case–control discrimination, we treated the ANN-derived probability of gastric cancer (pANN) as a continuous risk index in TCGA-STAD. Higher pANN values were associated with shorter overall survival (P = 0.006; **Supplementary Figure 2B**), and patients stratified by the median ANN score exhibited significantly divergent survival curves. This observation suggests that the expression patterns exploited by the ANN for diagnostic classification also reflect inter-patient heterogeneity in long-term outcome, even though SHAP values are not designed as direct surrogates of clinical hazard.

### 3.10 Validation of specific expression of network-prioritized genes in TME in human GC samples

**Figure 10:**
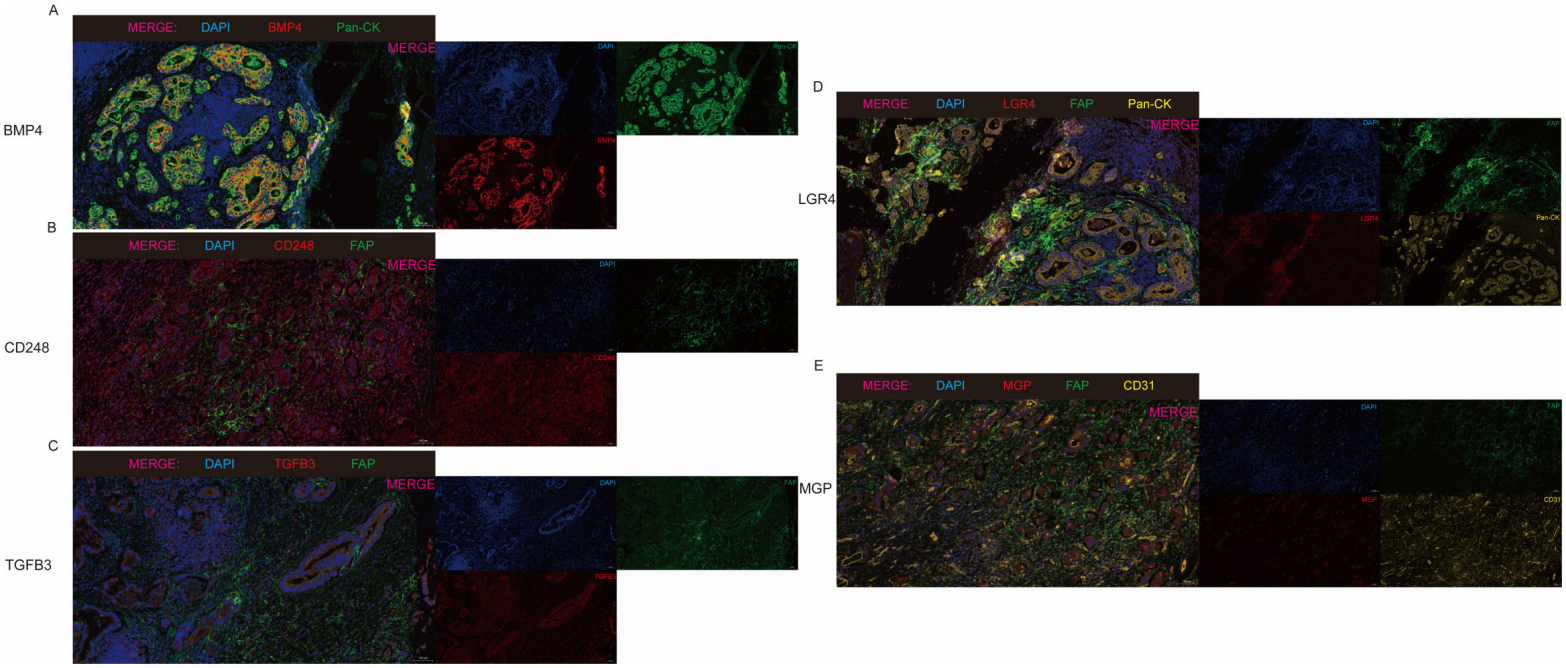
Multiplex immunofluorescence validation of hub gene expression in the gastric cancer tumor microenvironment. **(A)** Representative staining of BMP4 (red) and Pan-CK (green) in gastric cancer showing predominant localization of BMP4 to Pan-CK^+^ tumor epithelial nests. **(B)** CD248 (red) colocalizes with FAP^+^ cancer-associated fibroblasts (CAF; green) within the stromal compartment. **(C)** TGFB3 (red) is enriched in the FAP^+^ CAF-rich stroma. **(D)** LGR4 (red) is detected both in FAP^+^ CAFs (green) and in Pan-CK^+^ tumor epithelial cells (yellow). **(E)** MGP (red) is present in CD31^+^ endothelial structures (yellow) and in FAP^+^ CAF-rich stromal regions (green). Nuclei are counterstained with DAPI (blue). Left panels show merged images; right panels display the corresponding single-channel views. Scale bars, 100 μm. Integrating the proteome-wide MR screen with PPI topology, survival analysis, and compartment-resolved expression patterns, we prioritized ERBB3, LGR4, BMP4, CD248, MGP, TGFB3, GRP, and ETS2 as network-prioritized candidates for downstream contextualization. Among them, TGFB3 was selected for focused computational and cellular characterization on the basis of several convergent prioritization features, including its nominal MR association, network-topology ranking, association with overall survival, fibroblast-enriched expression pattern, and spatial organization in gastric cancer tissues. These observations provided a rationale for additional investigation. Accordingly, subsequent virtual screening, molecular docking, molecular dynamics simulation, and recombinant-TGFB3 perturbation experiments were performed as exploratory analyses to evaluate structural plausibility and cellular responses associated with TGFB3 exposure.

To orthogonally validate our transcriptomic findings, we performed multiplex immunofluorescence staining. Because ERBB3 and ETS2 emerged as putative protective factors in our transcriptomic analyses and GRP showed minimal expression within the gastric tumor microenvironment, we restricted the spatial validation to five oncogenic network-prioritized genes (BMP4, CD248, TGFB3, LGR4 and MGP). Multiplex immunofluorescence revealed that BMP4 protein predominantly colocalized with Pan-CK^+^ epithelial cells (**Figure 10A**). CD248 and TGFB3 signals extensively overlapped with FAP^+^ cancer-associated fibroblasts (CAFs), delineating a CAF-rich stromal compartment (**Figures 10B, C**). LGR4 was strongly enriched not only in Pan-CK^+^ epithelial cells but also within the FAP^+^ CAF compartment (**Figure 10D**), whereas MGP was detected in both CD31^+^ endothelial structures and the FAP^+^ CAF-rich stroma (**Figure 10E**). Collectively, these spatial localization patterns closely mirrored the cell type–specific expression profiles inferred from our transcriptomic analyses.

### 3.11 Virtual screening of candidate compounds from the ZINC database

In this study, we established a TGFB3-targeted virtual screening workflow using the ZINC database. Compounds were subjected sequentially to LigPrep ligand preparation, AutoDock Vina setup, an initial screen in Schrödinger Maestro, and high-precision Glide docking. Over 1,500 commercially available, drug-like small molecules were evaluated, and five compounds achieved Glide GScore values below –8.3 kcal/mol (**Table 1**).

**Table 1:** Top-ranked candidate compounds identified by ZINC virtual screening against TGFB3.

Name	Glide GScore (kcal/mol)
Proflavine hemisulfate	-9.323
Hydroxychloroquine sulfate	-9.215
Rizatriptan benzoate	-8.845
L-Histidine	-8.378
Retigabine	-8.327

More negative Glide GScores indicate stronger predicted binding affinity to TGFB3.

**Figure 11:**
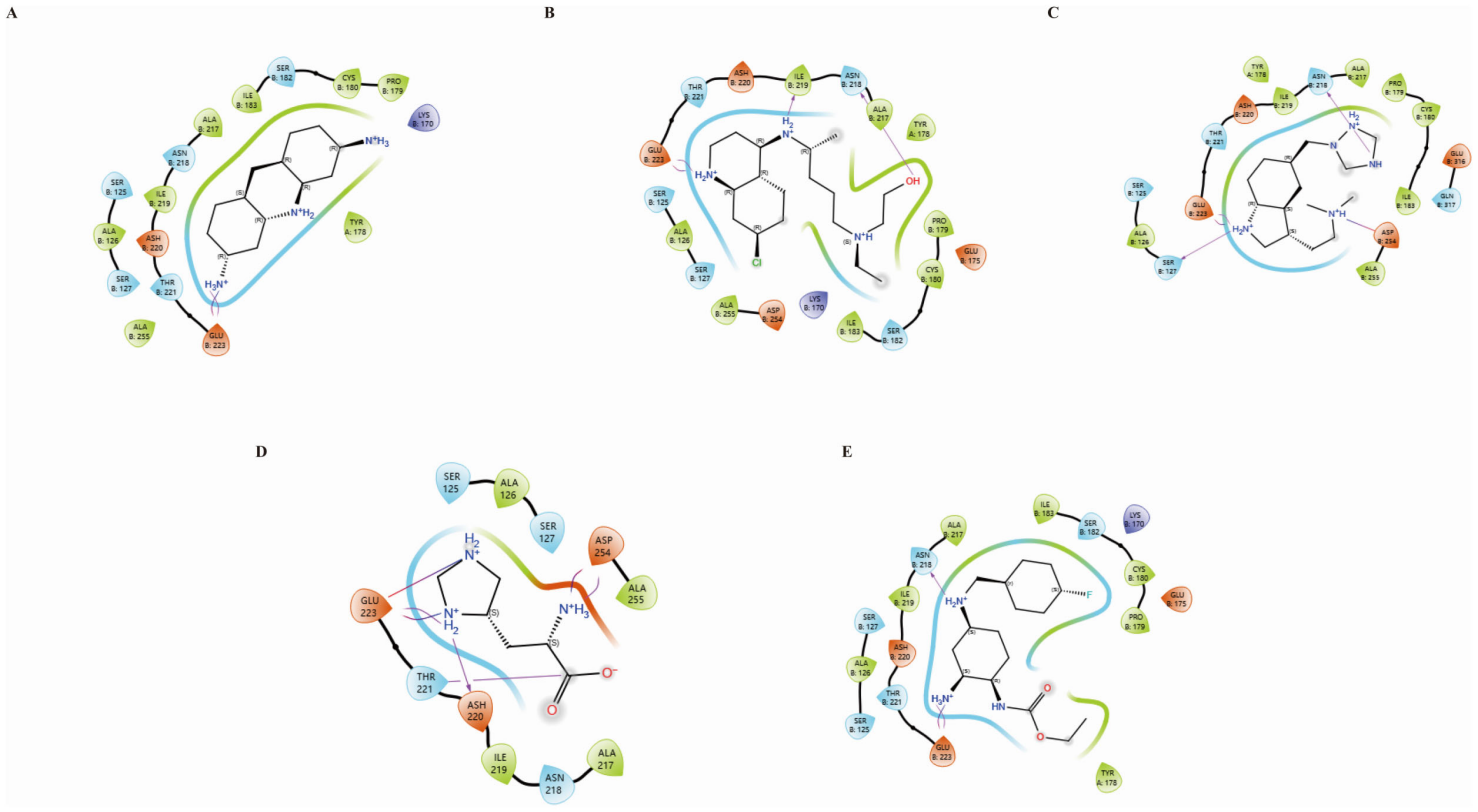
Two-dimensional chemical structures of the top five candidate compounds. **(A)** Proflavine hemisulfate. **(B)** Hydroxychloroquine sulfate. **(C)** Rizatriptan benzoate **(D)** L-Histidine. **(E)** Retigabine.

Of the five top−ranked compounds, ZINC000001530652 (Proflavine hemisulfate) exhibited the most favorable binding energy (–9.323 kcal/mol). Within the TGFB3 binding pocket, proflavine forms π–π stacking interactions with Tyr178 and Cys180 and establishes a bifurcated hydrogen−bond network with Glu223, Thr221 and Ser127 (**Figure 11A**). ZINC000003775644 (Hydroxychloroquine sulfate), ranked second (–9.215 kcal/mol), engages in multiple stable hydrogen bonds with Glu223, Thr221 and Asn218 and makes extensive hydrophobic contacts via its cyclic scaffold with Ile219, Ala217 and Ser125/127 (**Figure 11B**). The third−ranked ZINC000000005895 (Rizatriptan benzoate) docks at –8.845 kcal/mol, mediating hydrophobic and π–π interactions between its macrocyclic core and Phe221, Ile183 and Pro179, while concurrently forming hydrogen bonds with Glu223 and Asp254 (**Figure 11C**). Both the fourth−ranked ZINC000006661227 (L-Histidine; –8.378 kcal/mol; **Figure 11D**) and the fifth−ranked ZINC000000016154 (Retigabine; –8.327 kcal/mol; **Figure 11E**) establish two to three key hydrogen bonds within the active site and achieve high complementarity through polar side−chain interactions with adjacent polar and hydrophobic residues. These docking results provide ideal candidates for subsequent molecular dynamics simulations and form a robust theoretical foundation for TGFB3−targeted small−molecule drug design.

### 3.12 Molecular docking prediction of TGFB3 binding to proflavine hemisulfate, hydroxychloroquine sulfate, rizatriptan benzoate, L-histidine and retigabine

**Figure 12:**
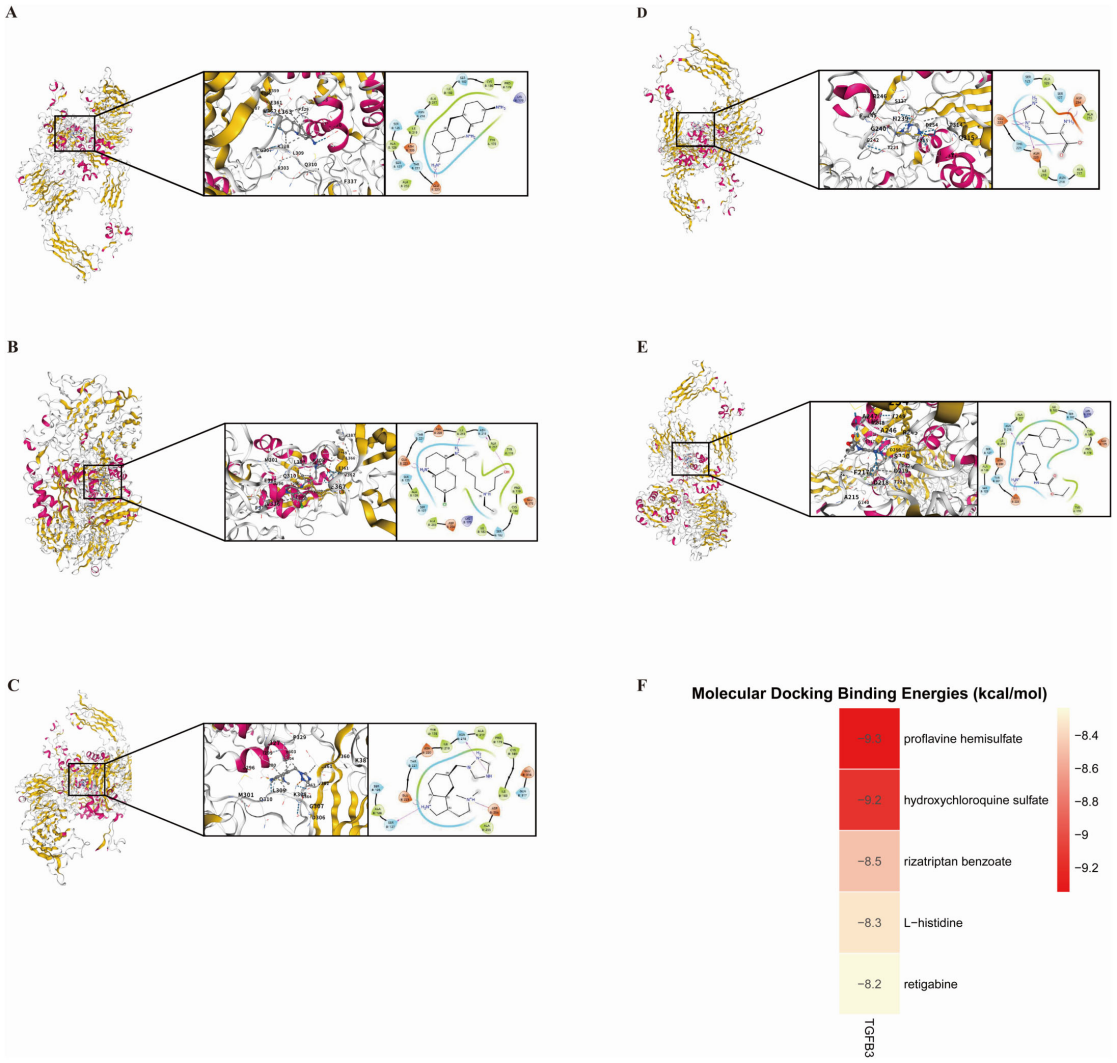
Molecular docking of TGFB3 with candidate ligands. The left panel in each subfigure depicts the three-dimensional ligand orientation within the TGFB3 binding pocket; the right panel presents a two-dimensional interaction diagram, highlighting hydrogen bonds and hydrophobic contacts. **(A)** Docking pose of TGFB3 with proflavine hemisulfate. **(B)** Docking pose of TGFB3 with hydroxychloroquine sulfate. **(C)** Docking pose of TGFB3 with rizatriptan benzoate. **(D)** Docking pose of TGFB3 with L-histidine. **(E)** Docking pose of TGFB3 with retigabine. **(F)** Heatmap of binding affinities (kcal/mol) obtained from molecular docking analyses between TGFB3 and the five ligands (proflavine hemisulfate, hydroxychloroquine sulfate, rizatriptan benzoate, L-histidine and retigabine).

To investigate the interactions between TGFB3 and five candidate ligands—proflavine hemisulfate, hydroxychloroquine sulfate, rizatriptan benzoate, L-histidine, and retigabine—we conducted molecular docking using AutoDock Vina. Each ligand was successfully accommodated within the TGFB3 binding domain, yielding binding affinities of –9.346, –9.153, –8.489, –8.324 and –8.234 kcal/mol, respectively. In the context of docking energy thresholds, values below –4.25 kcal/mol denote measurable binding activity, below –5.0 kcal/mol indicate good binding, and below –7.0 kcal/mol reflect very strong binding (**Figures 12A–F**). These results demonstrate that all five compounds exhibit robust binding interactions with TGFB3.

Of the five compounds identified through virtual screening and molecular docking—hydroxychloroquine sulfate, proflavine hemisulfate, rizatriptan benzoate, L-histidine and retigabine—proflavine hemisulfate demonstrated the lowest binding energy, indicating the most thermodynamically favorable interaction with TGFB3. Consequently, we selected the TGFB3–proflavine hemisulfate complex for all-atom molecular dynamics simulations under explicit solvent conditions. This approach will enable detailed assessment of the complex’s conformational stability, key residue interactions and dynamic behavior, thereby providing mechanistic insight into the potential inhibitory activity of proflavine hemisulfate against TGFB3.

### 3.13 Validation of binding stability and intermolecular interactions for the TGFB3–proflavine hemisulfate complex

**Figure 13:**
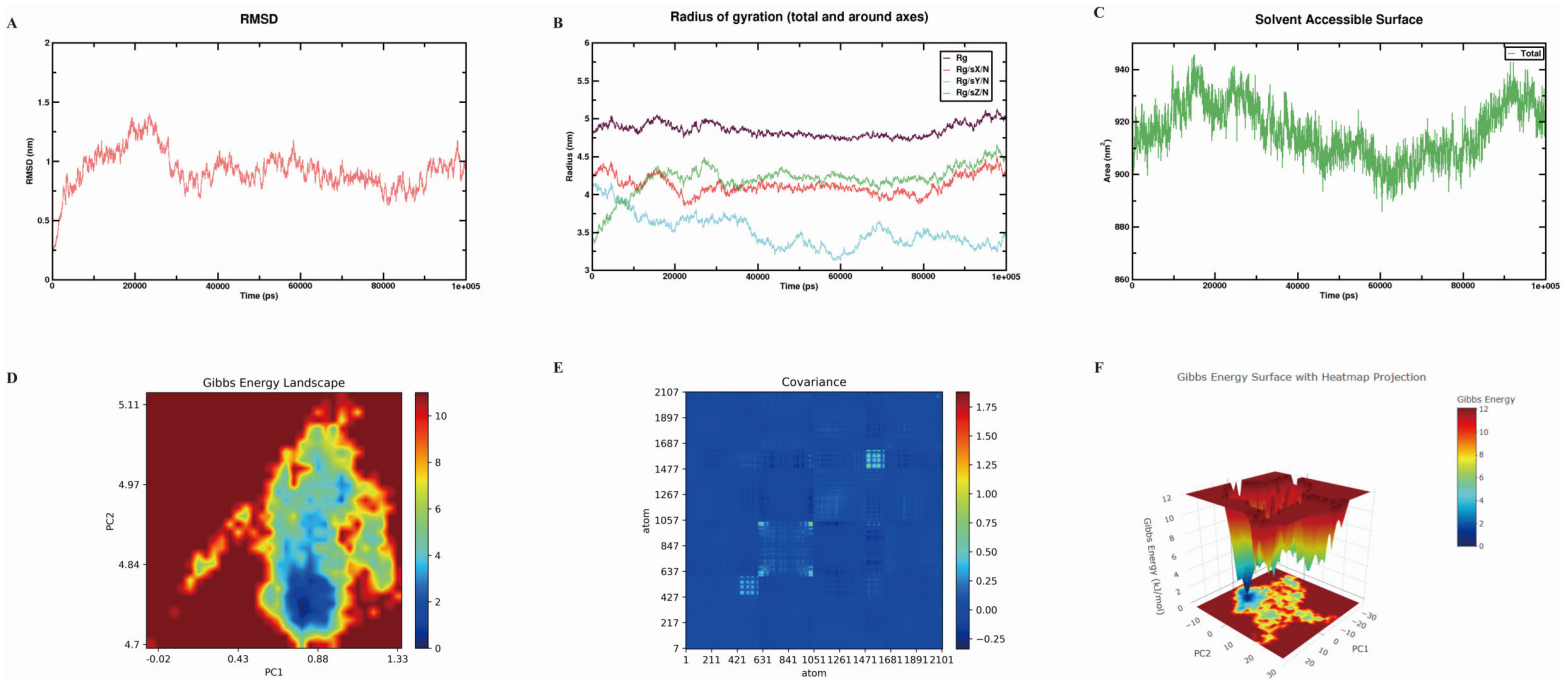
Molecular dynamics analysis of the TGFB3–proflavine hemisulfate complex over 100 ns. **(A)** Temporal evolution of the RMSD of the protein–ligand complex. **(B)** Time-dependent changes in the overall Rg and its axis-specific components (Rg_x, Rg_y, Rg_z). **(C)** SASA as a function of simulation time. **(D)** Two-dimensional Gibbs free energy landscape derived from principal component analysis (PC1–PC2), with the color gradient indicating free energy magnitude (kJ/mol). **(E)** Covariance matrix heatmap of atomic motions, where color intensity reflects the strength and direction (positive or negative) of residue–residue correlations. **(F)** Three-dimensional Gibbs free energy surface and its projected contour map on the PC1/PC2 plane, visually depicting the energy distribution along the principal components.

To assess the binding stability of the TGFB3–proflavine hemisulfate complex and its impact on protein conformational dynamics, we performed 100-ns (100,000-ps) molecular dynamics simulations in GROMACS. Trajectory analyses comprised RMSD, Rg, SASA, principal component analysis (PCA)–derived free energy landscapes, atomic motion covariance matrices, and three-dimensional free energy surfaces. The complex RMSD rose rapidly during equilibration and converged after ~20 ns, thereafter fluctuating narrowly between 0.20 and 0.30 nm, indicative of system equilibration and structural stability (**Figure 13A**). The Rg remained between 4.75 and 5.12 nm without substantial axis-specific deviations, reflecting a consistently compact tertiary fold (**Figure 13B**). SASA values were maintained at approximately 900–940 nm² throughout the simulation, suggesting that ligand binding did not appreciably alter the protein’s solvent-exposed surface (**Figure 13C**). The two-dimensional PCA free energy plot revealed two principal low-energy basins centered at PC1 ≈ 0.5 and PC2 ≈ 4.9 (ΔG ≈ 2–4 kJ/mol), indicating conformational sampling within discrete energetic minima (**Figure 13D**). The covariance heatmap demonstrated pronounced positive and negative correlations among residues lining the binding pocket, further substantiating proflavine-induced local dynamic stabilization (**Figure 13E**). Finally, the three-dimensional free energy surface and its projected contours along PC1/PC2 axes recapitulated these low-energy wells, underscoring the existence of a dominant stable conformation (**Figure 13F**). Collectively, these multidimensional analyses confirm that the TGFB3–proflavine hemisulfate complex maintains conformational integrity and exhibits ligand-stabilized dynamics throughout 100 ns of simulation and further suggest that proflavine hemisulfate may represent a promising therapeutic candidate for gastric cancer.

### 3.14 PFH effectively inhibits the proliferation of AGS gastric cancer cells

**Figure 14:**
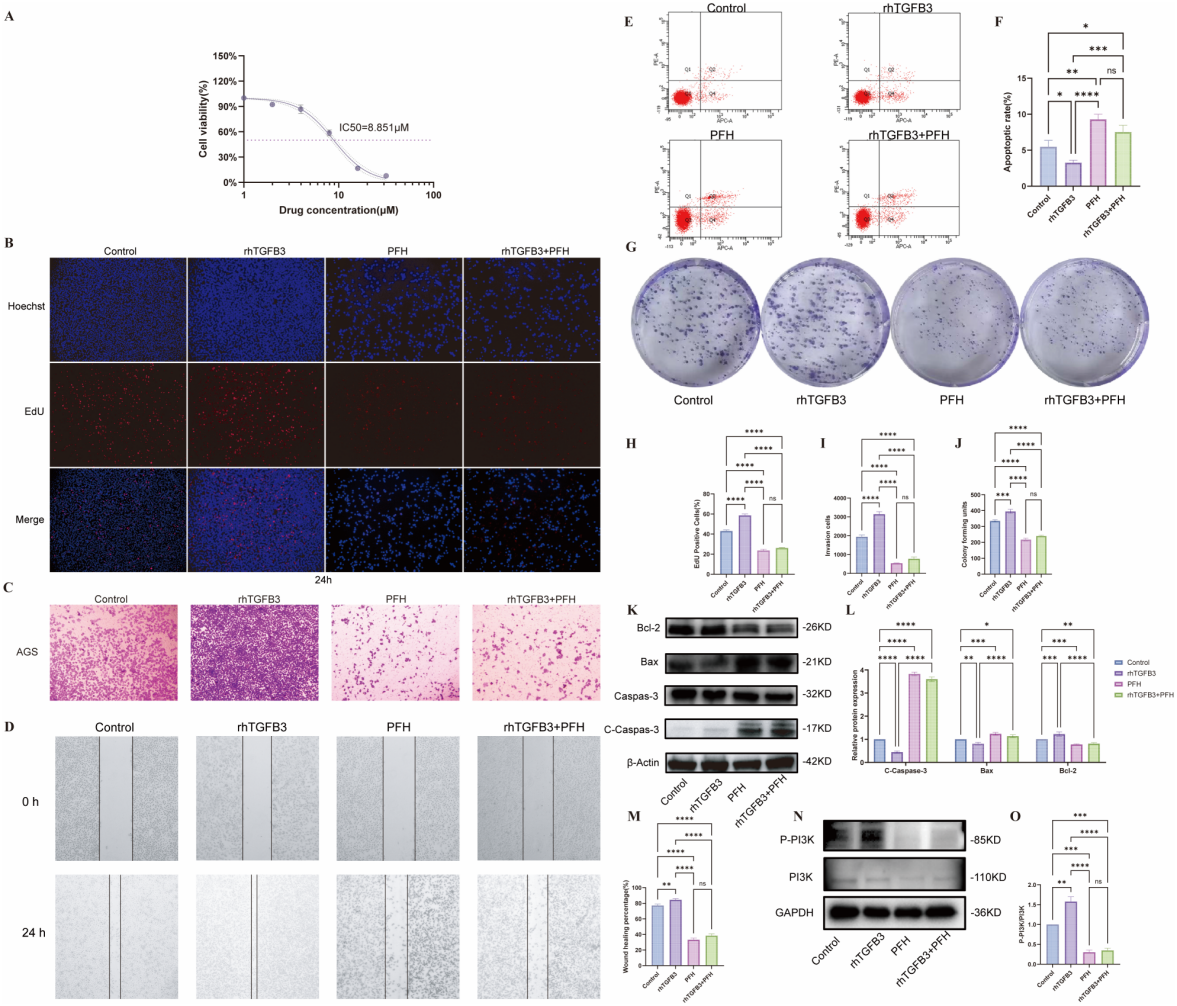
Proflavine hemisulfate suppresses proliferative and metastatic phenotypes while inducing apoptosis in AGS gastric cancer cells. **(A)** Cell viability of AGS cells treated for 24 h with increasing concentrations of proflavine hemisulfate (PFH), assessed by CCK-8 assay. **(B)** Representative EdU incorporation images of AGS cells exposed for 24 h to vehicle control, recombinant human TGFβ3 (rhTGFB3), PFH, or rhTGFB3 + PFH. EdU-positive (proliferating) nuclei are shown in red and all nuclei are counterstained with Hoechst (blue). **(C)** Representative Matrigel Transwell images showing AGS cells that invaded through the membrane under the indicated treatments. **(D)** Representative wound-healing images of AGS monolayers at 0 and 24 h after scratching in the four treatment groups. **(E)** Representative flow-cytometry plots of Annexin V/PI staining depicting apoptotic AGS cells after 24 h of treatment. **(F)** Quantification of the percentage of apoptotic cells derived from flow-cytometry analyses. **(G)** Representative colony-formation images of AGS cells cultured for 7 days under the indicated conditions. **(H)** Quantification of EdU-positive nuclei. **(I)** Quantification of invaded cells. **(J)** Quantification of colony numbers. **(K)** Representative immunoblots of apoptosis-related proteins (cleaved Caspase-3, Caspase-3, Bax and BCL2) with β-actin as a loading control in AGS cells treated for 24 h as indicated. **(L)** Densitometric analysis of apoptosis-related proteins normalized to β-actin. **(M)** Quantification of wound closure, expressed as the percentage of the initial wound area at 0 h. **(N)** Representative immunoblots of PI3K and phosphorylated PI3K (p-PI3K) with GAPDH as a loading control. **(O)** Quantification of the p-PI3K/PI3K ratio in the four groups. Data are presented as mean ± SD (n = 3 independent experiments). P values < 0.05 were considered statistically significant (*P < 0.05; **P < 0.01; ***P < 0.001; ****P < 0.0001).

We used a CCK-8 colorimetric assay to evaluate the effect of proflavine hemisulfate (PFH) on AGS cell viability. PFH induced a clear, dose-dependent decrease in cell viability, with a 24-h half-maximal inhibitory concentration (IC_50_) of 8.851 μM. On the basis of this dose–response relationship, we selected 8.851 μM PFH as the working concentration for all subsequent functional assays (**Figure 14A**). For rhTGFB3, we adopted a working concentration of 10ng/mL in accordance with previously published studies using gastric cancer cell lines, providing a biologically relevant reference condition for our experiments. To further substantiate the antiproliferative effect of PFH, we performed EdU incorporation and colony formation assays. The EdU assay revealed that PFH treatment markedly reduced the proportion of proliferating cells, as reflected by a decreased fraction of EdU-positive nuclei (**Figures 14B, H**). Consistently, colony formation assays demonstrated that PFH suppressed long-term clonogenic growth, leading to a significant reduction in both the number and size of colonies (**Figures 14G, J**). Together, these findings indicate that PFH effectively attenuates the proliferative capacity of AGS gastric cancer cells.

### 3.15 PFH attenuates the migratory and invasive capacities of AGS cells while promoting apoptosis

Next, we investigated whether PFH modulates the metastatic traits and survival of AGS cells. In wound-healing assays, PFH treatment markedly delayed closure of the scratch area, indicating a pronounced impairment of lateral migratory capacity (**Figures 14D, M**). Consistently, Transwell invasion assays showed that PFH significantly reduced the number of cells traversing the Matrigel-coated inserts (**Figures 14C, I**). Concordant with these anti-motility effects, Annexin V/PI–based flow cytometric analysis revealed that PFH treatment substantially increased the proportion of apoptotic AGS cells (**Figures 14E, F**). Taken together, these findings demonstrate that PFH not only weakens the migratory and invasive behavior of AGS gastric cancer cells but also promotes apoptosis, supporting its potential to limit metastatic progression.

### 3.16 PFH shifts the apoptotic balance and suppresses TGFB3-induced PI3K activation in AGS cells

Western blotting further confirmed that PFH engages the apoptotic machinery and counteracts TGFB3-driven survival signaling in AGS cells. Compared with the control group, rhTGFB3 treatment reduced the levels of cleaved Caspase-3 and Bax while increasing the expression of the anti-apoptotic protein Bcl-2, consistent with a pro-survival shift in the balance of Bcl-2 family members. In contrast, PFH markedly enhanced cleaved Caspase-3 and Bax and concomitantly decreased Bcl-2 expression, and co-treatment with PFH largely reversed the rhTGFB3-induced changes (**Figures 14K, L**). These data indicate that PFH skews the apoptotic rheostat towards cell death and effectively antagonizes the anti-apoptotic influence of TGFB3.

To interrogate whether this effect is linked to modulation of PI3K signaling, we next examined PI3K phosphorylation. In serum-cultured control cells, a low but detectable level of p-PI3K relative to total PI3K was observed, reflecting tonic PI3K activity required for basal proliferation and survival. Exposure to rhTGFB3 markedly increased the p-PI3K/PI3K ratio without altering total PI3K abundance, confirming robust activation of the PI3K pathway. By contrast, PFH substantially decreased PI3K phosphorylation, both under basal conditions and in the presence of rhTGFB3, while total PI3K and GAPDH levels remained essentially unchanged (**Figures 14N, O**). Thus, PFH does not simply cause nonspecific protein loss or global cytotoxic damage but instead selectively attenuates TGFB3-induced PI3K activation. Together, these findings support a model in which PFH promotes apoptosis of AGS gastric cancer cells at least in part by dampening PI3K-dependent survival signaling.

### 3.17 Computational characterization of a putative fibroblast–epithelial TGFB3 signaling route

**Figure 15:**
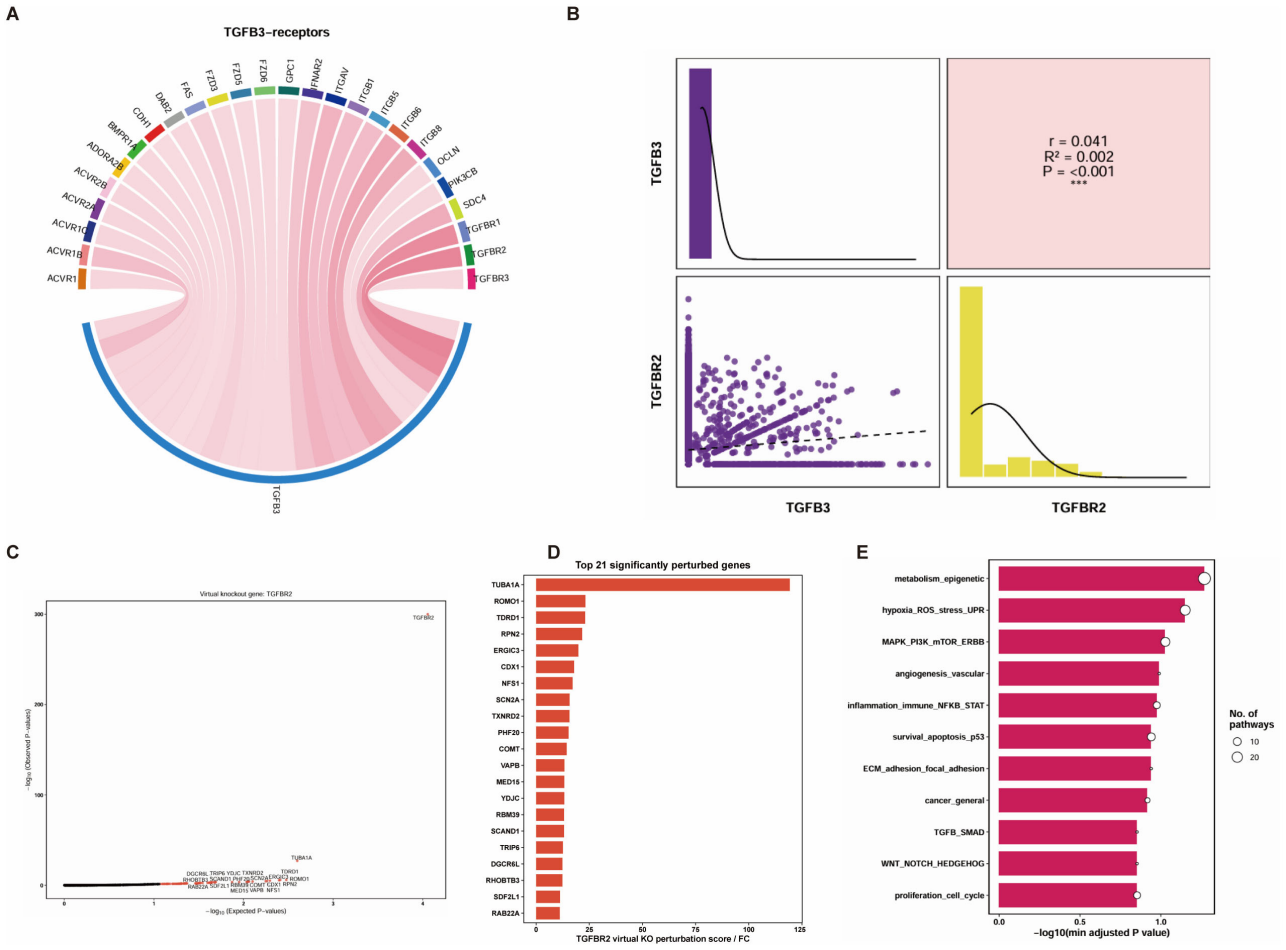
**(A)** NicheNet ligand–receptor analysis using fibroblasts as sender cells and epithelial cells as receiver cells, with TGFB3 specified as the ligand of interest. Candidate receptors associated with TGFB3 are displayed. **(B)** Scatter plot showing the relationship between TGFB3 and TGFBR2 expression. The Spearman correlation coefficient and corresponding P value are indicated in the panel. **(C)** Manifold-alignment plot generated after in silico knockout of TGFBR2 in epithelial cells using scTenifoldKnk. Each point represents one gene and selected genes are labeled. **(D)** Ranking of genes affected following virtual TGFBR2 knockout according to the perturbation statistics generated by scTenifoldKnk. **(E)** Functional enrichment analysis result of genes affected by virtual TGFBR2 knockout.

Single-cell expression patterns localized TGFB3 predominantly to the fibroblast compartment, suggesting that its potential effects on malignant epithelial cells may involve paracrine rather than epithelial cell-autonomous signaling. Consistent with this possibility, fibroblast-to-epithelial NicheNet analysis placed TGFB3 within a set of plausible ligand–receptor relationships and identified TGFBR2 as one candidate epithelial receptor associated with TGFB3-linked signaling (**Figure 15A**). Cell-level Spearman analysis showed a statistically significant positive association between TGFB3 and TGFBR2 expression (Spearman ρ = 0.041, P < 0.001; **Figure 15B**). Virtual knockout of TGFBR2 in epithelial cells using scTenifoldKnk identified a set of transcriptionally perturbed genes (**Figures 15C, D**). Functional enrichment of the perturbed gene set implicated biological themes related to TGF-β/SMAD signaling, MAPK–PI3K–mTOR–ERBB signaling, proliferation and cell-cycle regulation, survival–apoptosis–p53 signaling, extracellular-matrix and focal-adhesion processes, angiogenesis, and inflammatory signaling (**Figure 15E**). Together, these analyses provide computational support for a putative fibroblast-derived TGFB3–TGFBR2-linked signaling route in epithelial cells.

## 4 Discussion

In this study, we applied a proteome-wide Mendelian randomization framework to systematically evaluate associations between genetically predicted circulating levels of 4,907 plasma proteins and gastric cancer risk. 29 proteins met the prespecified discovery criterion, from which eight candidates—ERBB3, LGR4, BMP4, CD248, MGP, TGFB3, GRP, and ETS2—were further prioritized through network topology and complementary multi-omics analyses. Accordingly, the downstream transcriptomic, spatial, network, and experimental analyses in this study were used to contextualize and prioritize the MR signals.

Consistent with published gastric cancer studies, all eight MR-prioritized proteins show independent biological support in gastric tumorigenesis. In gastric cancer, ERBB3 (HER3) is typically overexpressed at the tumor cell membrane and has been linked to advanced stage, therapeutic resistance and poor survival, supporting a predominantly oncogenic role in this setting (41, 42). By contrast, in bladder cancer higher circulating/soluble HER3 levels predict improved overall survival, indicating that non-membrane HER3 may reflect a compensatory or host-protective response rather than direct tumor promotion (43). Our MR findings are concordant with this latter pattern, as genetically predicted higher circulating ERBB3 levels were associated with a reduced risk of gastric cancer, highlighting the compartment- and context-dependent nature of HER3 biology and underscoring the need for further mechanistic studies. ETS2, a transcription factor with established tumor-suppressive properties in several malignancies, has also been implicated in gastric cancer where its reduced expression correlates with aggressive phenotypes and adverse outcome, presumably through dysregulation of cell-cycle and apoptosis programs (44). By contrast, the remaining six proteins (BMP4, CD248, GRP, LGR4, MGP and TGFB3) converge on pro-tumorigenic pathways that are highly relevant to gastric cancer biology. BMP4, a canonical ligand in the TGF-β superfamily, can activate SMAD-dependent signaling, promote epithelial–mesenchymal transition and enhance migration and invasion of gastric cancer cells (45). CD248 (endosialin/TEM1), a marker of cancer-associated fibroblasts and perivascular stromal cells, contributes to extracellular-matrix remodeling, angiogenesis and an immunosuppressive microenvironment in gastric tumors (46, 47). GRP functions as an autocrine/paracrine neuropeptide growth factor; overexpression of GRP and its receptor has been reported in gastric and other gastrointestinal cancers and is linked to increased proliferation, motility and metastatic potential (48). LGR4, a receptor for R-spondins that potentiates Wnt/β-catenin signaling, is upregulated in gastric cancer tissues and has been associated with enhanced stemness, invasion and poor prognosis (49, 50). MGP (matrix Gla protein), an extracellular matrix–associated protein, has been found to correlate with tumor progression, neovascularization and stromal activation in gastrointestinal malignancies, including gastric cancer (51). TGFB3, a member of the TGF-β isoform family, orchestrates cell-cycle arrest, apoptosis evasion, immune suppression and EMT; aberrant activation of TGF-β signaling and elevated TGFB3 expression in gastric cancer are frequently associated with advanced stage (52).Using the eight network-prioritized genes, we developed an ANN classifier that showed reproducible discrimination between gastric cancer and non-tumor tissues across the evaluated cohorts. The apparent training AUC of 0.998 was not considered the primary estimate of generalizability because it was calculated in the model-development dataset and may reflect optimism. More conservative estimates were obtained through repeated cross-validation and bootstrap optimism correction in TCGA-STAD, yielding AUCs of 0.913 and 0.908, respectively. The locked TCGA-trained model retained discriminatory performance in the independent GSE54129, GSE26899, and GSE13911 cohorts, with AUCs of 0.953, 0.837, and 0.819, respectively. Preprocessing, feature scaling, and hyperparameter tuning were performed within the internal resampling procedure, whereas no retraining, feature reselection, or hyperparameter optimization was conducted in the external cohorts. The five-neuron hidden layer was selected from a prespecified tuning grid on the basis of repeated cross-validated performance rather than chosen arbitrarily.

Direct comparisons using the same eight-gene panel and validation framework showed that the ANN achieved the strongest overall discrimination relative to standard logistic regression, ridge logistic regression, LASSO logistic regression, and random forest. However, the competitive performance of simpler models, particularly LASSO logistic regression, indicates that a substantial proportion of the diagnostic signal is attributable to the eight-gene feature set itself rather than exclusively to the neural-network architecture. The ANN should therefore be regarded as the best-performing implementation under the present analytical framework, rather than as a universally superior classification method. Seed-stability analysis across 100 random initializations showed stable median performance, with cross-validated and bootstrap-corrected AUCs of 0.910 and 0.905, respectively, and the final locked model performed close to these central estimates. Nevertheless, occasional low-performing initializations indicate residual sensitivity to model convergence and random weight initialization. Although the ANN-derived score remained statistically informative after adjustment for available clinicopathological variables, its incremental clinical value requires prospective evaluation. In addition, PPV and NPV were treated only as cohort-level descriptive measures because they are prevalence-dependent and cannot be directly extrapolated from retrospective case–control datasets to population screening settings. Future studies should compare the ANN prospectively with simpler transcriptomic models and established biomarkers such as CEA and CA19–9 using clinically representative disease prevalence and prespecified decision thresholds.

Given the inherent opacity of neural networks, we applied SHAP to improve interpretability and quantify the contribution of each hub gene to ANN predictions. Globally, SHAP analysis established a consistent hierarchy of feature importance, with CD248, ERBB3 and LGR4 emerging as major contributors, while dependence and beeswarm plots showed that individual genes can exert positive or negative effects on the predicted probability of gastric cancer depending on their expression context, consistent with non-linear and interaction-dependent decision boundaries. We explicitly interpret SHAP values as descriptors of model behavior rather than as estimates of biological or causal risk: comparisons with single-gene Cox hazard ratios revealed no simple monotonic relationship between SHAP importance and marginal survival effects. Nonetheless, the ANN-derived probability score itself was associated with overall survival, indicating that the transcriptional patterns exploited for diagnostic classification also capture prognostic heterogeneity. Stability analyses across multiple random initializations and bootstrap resampling further demonstrated that both the ANN predictions and their SHAP-based explanations are robust to stochastic variation and sampling noise.

Single-cell analysis provided cellular context for the prioritized genes. Reference-based SingleR annotation supported the broad marker-based lineage assignments, and formal Wilcoxon testing identified cell-type-associated differences in expression. ERBB3 was predominantly localized to epithelial cells, whereas CD248, MGP, TGFB3, LGR4, and BMP4 showed varying degrees of stromal or endothelial expression.

CellChat identified statistically supported, expression-based ligand–receptor relationships among epithelial, immune, stromal, and endothelial populations. The interaction-count network represented the number of inferred ligand–receptor pairs, whereas the interaction-weight network represented aggregated model-derived communication probabilities (30). The Monocle 2 trajectory analysis incorporated multiple biologically distinct lineages, it cannot reconstruct a common developmental pathway or a temporal sequence of gastric cancer progression. We therefore interpret the inferred axis as an exploratory transcriptional-state continuum (53). The Kruskal–Wallis analysis demonstrated substantial differences in pseudotime distributions among the eight cell types, and within-cell-type Spearman analyses identified associations between selected candidate genes and the numerical pseudotime axis. Spatial transcriptomic analysis provided additional evidence that the expression of the eight candidates was spatially structured. Global Moran’ s I demonstrated statistically significant positive spatial autocorrelation for all eight genes in both analyzed sections. This result indicates that expression values were distributed non-randomly across the tissue sections. Complementing these transcriptomic observations, multiplex immunofluorescence staining for a subset of hubs (TGFB3, CD248, MGP, LGR4 and BMP4) demonstrated concordant protein-level localization in malignant epithelium, CAF-rich stroma and perivascular regions, thereby providing orthogonal histopathological validation of their compartment-specific expression.

Among the eight candidates, TGFB3 was selected for additional investigation because of the convergence of several prioritization criteria, including its MR estimate, PPI network position, survival association, stromal expression, spatial organization, and suitability for structure-based computational analysis. TGF-β signaling is highly pleiotropic and can exert different, or even opposing, effects depending on the responding cell type, receptor availability, disease stage, and tissue context. The designation of TGFB3 as a downstream study candidate therefore reflects an analytical prioritization decision.

Using virtual screening of drug-like compounds from ZINC, followed by docking and molecular dynamics simulations, we identified five candidate TGFB3-binding ligands with favorable docking energies and stable interaction profiles, of which proflavine hemisulfate (PFH) displayed the most favorable binding energy and robust conformational stability in all-atom simulations. These methods provide structural plausibility and assess the stability of a predicted complex within a force-field model, but they do not directly establish biochemical binding affinity, selectivity, target engagement, or inhibition of TGFB3 signaling.

The *in vitro* experiments demonstrated that recombinant TGFB3 exposure was associated with changes in proliferation, migration, invasion, apoptosis, and PI3K-related signaling readouts in AGS cells and that PFH attenuated several of these responses under the tested conditions. These observations provide functional characterization of epithelial responses in an exogenous-ligand system.

NicheNet analysis identified a putative TGFB3–TGFBR2 signaling axis mediating intercellular communication between fibroblasts and epithelial cells. At the single-cell level, TGFB3 and TGFBR2 exhibited a statistically significant positive association and coordinated expression pattern, supporting the potential functional coupling of this ligand–receptor pair. Consistently, in silico perturbation of TGFBR2 using scTenifoldKnk revealed widespread transcriptional consequences involving TGF-β/SMAD signaling, MAPK–PI3K–mTOR–ERBB pathway activity, cell-cycle regulation, cell-survival programs, extracellular-matrix organization, cell adhesion, angiogenesis, and inflammatory responses.

At the same time, our findings underscore that PFH should be viewed as a chemical probe and repurposing lead rather than as an immediately translatable systemic therapeutic. In silico absorption, distribution, metabolism, excretion and toxicity (ADMET) predictions across multiple platforms suggest modest oral bioavailability, limited blood–brain barrier penetration and predominant hepatic clearance (**Supplementary Table 18**), but also flag non-trivial risks of mutagenicity and potential cardiotoxicity or hepatotoxicity at higher systemic exposures—consistent with the known DNA-intercalating properties and historical antimicrobial use of acriflavine-like dyes. Literature review likewise indicates that while PFH and its analogues can induce apoptosis and autophagy in tumor cells, their genotoxic potential constrains chronic systemic administration (54, 55). PFH may therefore serve as a preliminary scaffold or perturbagen for subsequent target-engagement, structure–activity, selectivity, pharmacokinetic, and toxicological studies. Direct biochemical binding assays, TGFB3-specific rescue experiments, comparison with structurally related analogues, and *in vivo* validation will be required before TGFB3 druggability or PFH-mediated target inhibition can be established.

Several limitations should be acknowledged. First, the proteome-wide MR analysis was exploratory. Although genome-wide significant instruments, LD clumping, Steiger filtering, instrument-strength assessment, and sensitivity analyses were applied, none of the 29 prioritized proteins met the Bonferroni threshold for 4,907 tests (**Supplementary Table 19**). Because individual-level plasma-protein covariance data were unavailable, the empirical correlation structure among proteins and permutation-based FDR could not be evaluated. Moreover, nonsignificant MR-Egger, MR-PRESSO, Cochran’s Q, and leave-one-out results reduce concern regarding major instrument-related bias but do not exclude horizontal pleiotropy, linkage disequilibrium, or weakly detectable heterogeneity. Second, the gastric cancer GWAS contained substantially fewer cases than controls, limiting precision for small or context-dependent associations. Plasma pQTLs were derived primarily from an Icelandic population, whereas the outcome datasets included European and East Asian populations. Differences in allele frequency, linkage disequilibrium structure, environmental exposure, and tumor composition may affect trans-ancestry transportability. Concordant effect directions across independent datasets support reproducibility but do not exclude ancestry-specific effects, which will require evaluation in larger multi-ancestry proteomic and gastric cancer GWAS. Third, the downstream multi-omics analyses remain subject to method-specific constraints. PPI centrality may be influenced by literature-density and database-annotation bias. The single-cell analysis included nine tumor specimens from six patients, limiting biological replication, and cell-level differential-expression and pseudotime analyses do not fully account for within-patient dependence. CellChat and NicheNet infer communication from expression patterns and curated prior networks rather than directly measuring ligand–receptor binding or signaling activity. The multi-lineage pseudotime trajectory represents an exploratory transcriptional-state continuum rather than a developmental or temporal sequence. Similarly, Global Moran’s I establishes gene-specific spatial autocorrelation but does not demonstrate inter-gene colocalization, histological-interface specificity, or functional interaction. These analyses therefore provide complementary contextualization and hypothesis generation rather than mechanistic validation. Finally, functional characterization was deliberately focused. TGFB3 was predominantly localized to fibroblasts, whereas the present experiments assessed responses of a single gastric cancer epithelial cell line to recombinant TGFB3. These assays demonstrate epithelial responsiveness under the tested conditions but do not establish that endogenous fibroblast-derived TGFB3 is necessary or sufficient for the observed phenotypes. Definitive evaluation will require fibroblast-specific TGFB3 perturbation, measurement of secreted TGFB3, fibroblast–epithelial co-culture or conditioned-medium experiments, rescue studies, and validation in organoid and *in vivo* models. Furthermore, molecular docking and molecular dynamics provide computational structural plausibility but do not demonstrate biochemical binding, selectivity, or cellular target engagement. Given its pleiotropic and DNA-intercalating properties, proflavine hemisulfate should be considered a preliminary pharmacological perturbagen rather than a selective TGFB3 probe or therapeutic candidate. Additional biochemical, pharmacokinetic, toxicological, and medicinal-chemistry studies are required before translational conclusions can be drawn.

## 5 Conclusion

In this study, we integrated proteome-wide Mendelian randomization, artificial neural network modeling, SHAP interpretation, single-cell and spatial transcriptomic analyses, virtual screening, molecular docking, molecular dynamics simulations, and *in vitro* assays to investigate the relationship between circulating plasma proteins and gastric cancer. This integrative framework prioritized eight candidates—ERBB3, LGR4, BMP4, CD248, MGP, TGFB3, GRP, and ETS2—with genetically informed associations and complementary expression, cellular, spatial, and clinical evidence. The eight-gene signature also showed reproducible diagnostic discrimination across the evaluated retrospective cohorts. Among these candidates, TGFB3 was selected for additional characterization, and proflavine hemisulfate attenuated selected phenotypic and signaling responses following recombinant TGFB3 exposure under the tested conditions. Collectively, these findings provide a genetically informed and multi-omics-supported set of candidate biomarkers and signaling factors for further diagnostic, prognostic, and therapeutic investigation in gastric cancer.

## Data Availability

The original contributions presented in the study are included in the article/**Supplementary Material**. Further inquiries can be directed to the corresponding authors.
