# Supplementary material for: From proteome-wide Mendelian randomization and multi-omics integration to functional validation: TGFB3 as a prioritized candidate in gastric adenocarcinoma
Source: Front Oncol. 2026 Jul 6;16:1883227. doi: 10.3389/fonc.2026.1883227 (PMC13381258; doi:10.3389/fonc.2026.1883227)
Supplement: Supplementary file 2 [file Image1.pdf]

Supplementary Figure 1

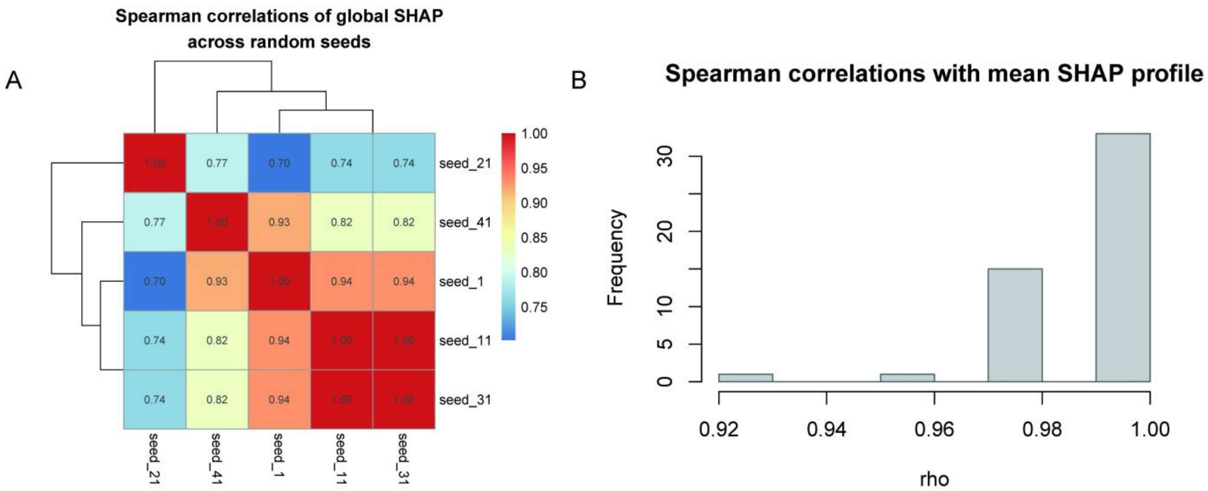

Figure 1. A.Heatmap of pairwise Spearman correlations (ρ) between global SHAP value profiles of the eight-gene panel obtained from five independently initialized neural networks (seeds 1, 11, 21, 31, and 41). B. Distribution of Spearman correlations between the global SHAP profiles from bootstrap resamples and the overall mean SHAP profile.
