# Supplementary material for: From proteome-wide Mendelian randomization and multi-omics integration to functional validation: TGFB3 as a prioritized candidate in gastric adenocarcinoma
Source: Front Oncol. 2026 Jul 6;16:1883227. doi: 10.3389/fonc.2026.1883227 (PMC13381258; doi:10.3389/fonc.2026.1883227)
Supplement: Supplementary file 3 [file Image2.pdf]

Supplementary Figure 2

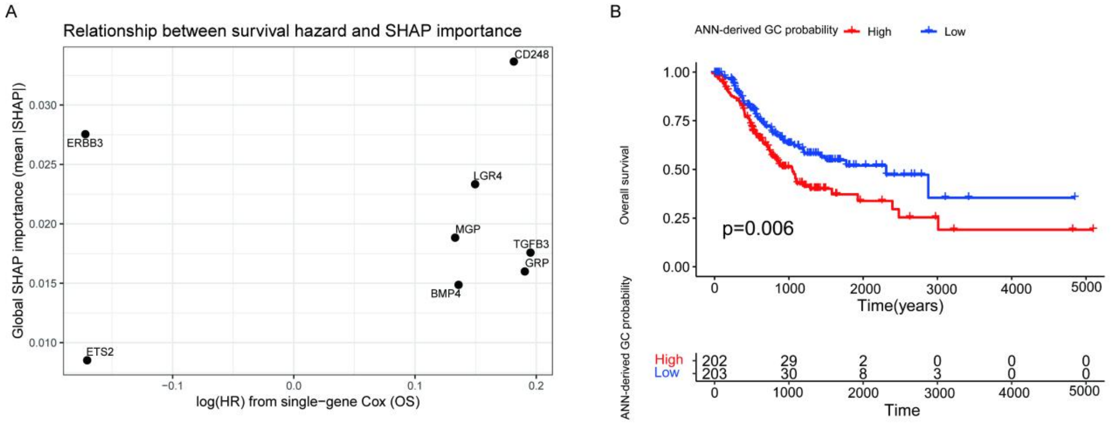

Figure 2. A. Scatter plot depicting the relationship between log hazard ratios from Cox regression for each individual gene and its global SHAP importance (mean |SHAP|). B. Kaplan–Meier curves for overall survival in patients stratified into high and low groups according to the ANN output (continuous predicted probability).
