# Supplementary material for: From proteome-wide Mendelian randomization and multi-omics integration to functional validation: TGFB3 as a prioritized candidate in gastric adenocarcinoma
Source: Front Oncol. 2026 Jul 6;16:1883227. doi: 10.3389/fonc.2026.1883227 (PMC13381258; doi:10.3389/fonc.2026.1883227)
Supplement: Supplementary file 4 [file Image3.pdf]

Supplementary Figure 3

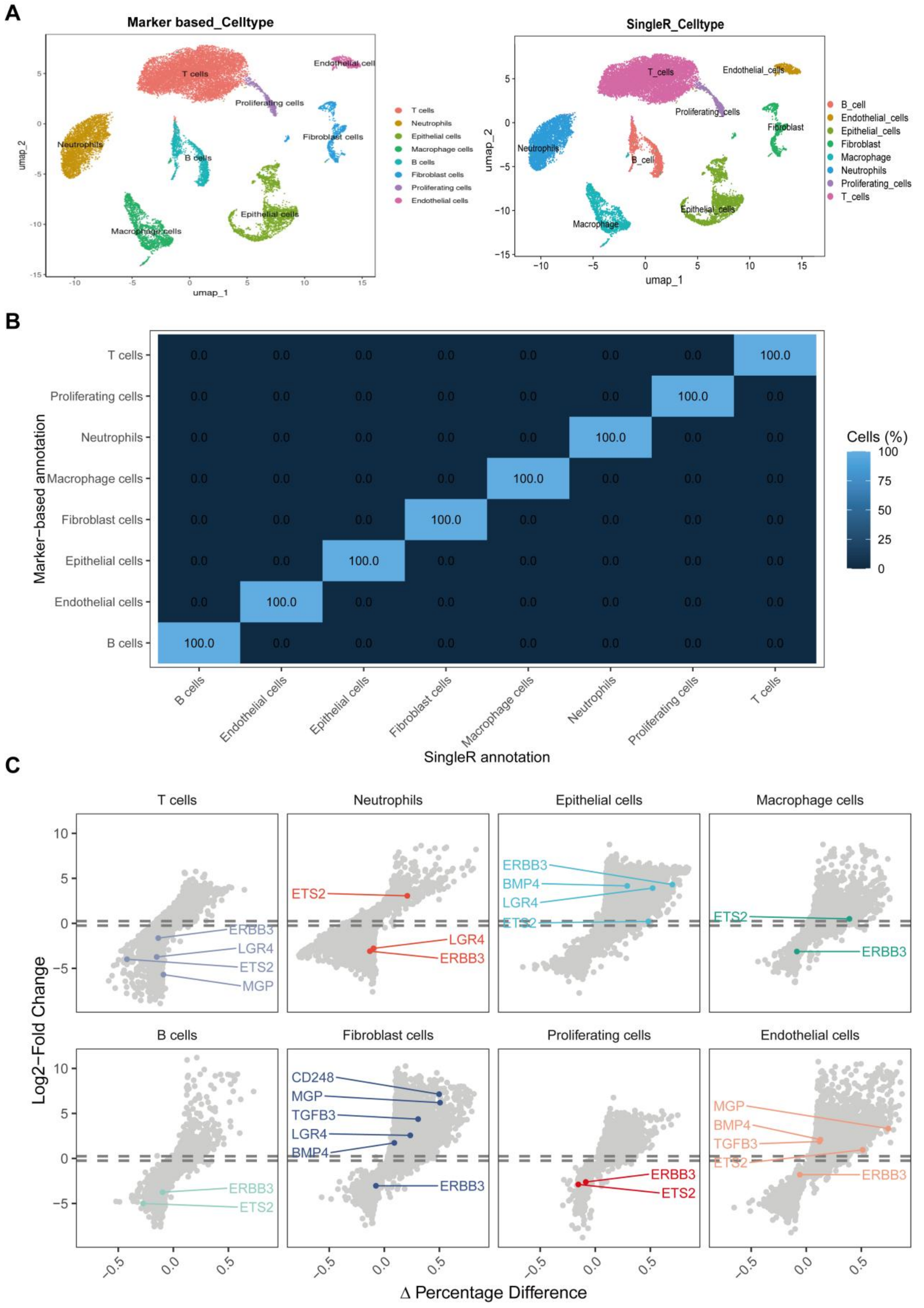

Figure 3. A. UMAP visualization of 20,491 high-quality cells obtained from nine gastric cancer specimens, colored according to marker-based annotations (left) and

SingleR-derived annotations (right). B. Concordance matrix comparing marker-based and SingleR-derived cell-type assignments after harmonization of annotation terminology. Rows represent marker-based annotations and columns represent SingleR annotations. Values indicate the percentage of cells within each marker-defined lineage assigned to the corresponding SingleR lineage. C. Cell-type-associated differential-expression effect-size plots generated from Wilcoxon rank-sum tests implemented in Seurat.
