# Supplementary material for: From proteome-wide Mendelian randomization and multi-omics integration to functional validation: TGFB3 as a prioritized candidate in gastric adenocarcinoma
Source: Front Oncol. 2026 Jul 6;16:1883227. doi: 10.3389/fonc.2026.1883227 (PMC13381258; doi:10.3389/fonc.2026.1883227)
Supplement: Supplementary file 5 [file Image4.pdf]

# Supplementary Figure 4

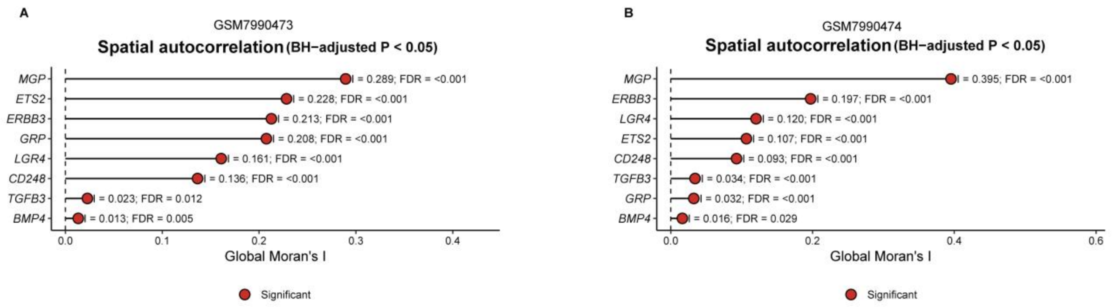


Figure 4. Global spatial autocorrelation of the eight network-prioritized genes in gastric cancer spatial transcriptomic sections. Global Moran's I statistics for ERBB3, BMP4, LGR4, CD248, MGP, TGFB3, GRP, and ETS2 were calculated separately in (A) GSM7990473 and (B) GSM7990474 using spot-level gene expression and a spatial-neighbor graph derived from the tissue coordinates. Genes are ordered according to their Global Moran's I values. Points indicate the Moran's I statistic, and horizontal segments extend from zero to the corresponding value. Text labels show the Moran's I statistic and Benjamini–Hochberg-adjusted P value for each gene.
